# Analysis of the role of the QseBC two-component sensory system in epinephrine-induced motility and intracellular replication of *Burkholderia pseudomallei*

**DOI:** 10.1371/journal.pone.0282098

**Published:** 2023-02-23

**Authors:** Chatruthai Meethai, Muthita Vanaporn, Narin Intarak, Varintip Lerdsittikul, Patoo Withatanung, Sujintana Janesomboon, Paiboon Vattanaviboon, Sorujsiri Chareonsudjai, Toby Wilkinson, Mark P. Stevens, Joanne M. Stevens, Sunee Korbsrisate

**Affiliations:** 1 Department of Immunology, Faculty of Medicine Siriraj Hospital, Mahidol University, Bangkok, Thailand; 2 Department of Microbiology and Immunology, Faculty of Tropical Medicine, Mahidol University, Bangkok, Thailand; 3 Department of Physiology, Faculty of Dentistry, Genomics and Precision, Chulalongkorn University, Bangkok, Thailand; 4 Microbiology Laboratory, Faculty of Veterinary Science, Veterinary Diagnostic Center, Mahidol University, Nakhon Pathom, Thailand; 5 Laboratory of Biotechnology, Chulabhorn Research Institute, Bangkok, Thailand; 6 Department of Microbiology, Faculty of Medicine, Khon Kaen University, Khon Kaen, Thailand; 7 The Roslin Institute and Royal (Dick) School of Veterinary Studies, University of Edinburgh, Midlothian, United Kingdom; University of Illinois at Urbana-Champaign, UNITED STATES

## Abstract

*Burkholderia pseudomallei* is a facultative intracellular bacterial pathogen that causes melioidosis, a severe invasive disease of humans. We previously reported that the stress-related catecholamine hormone epinephrine enhances motility of *B*. *pseudomallei*, transcription of flagellar genes and the production of flagellin. It has been reported that the QseBC two-component sensory system regulates motility and virulence-associated genes in other Gram-negative bacteria in response to stress-related catecholamines, albeit disparities between studies exist. We constructed and whole-genome sequenced a mutant of *B*. *pseudomallei* with a deletion spanning the predicted *qseBC* homologues (*bpsl0806* and *bpsl0807*). The Δ*qseBC* mutant exhibited significantly reduced swimming and swarming motility and reduced transcription of *fliC*. It also exhibited a defect in biofilm formation and net intracellular survival in J774A.1 murine macrophage-like cells. While epinephrine enhanced bacterial motility and *fliC* transcription, no further reduction in these phenotypes was observed with the Δ*qseBC* mutant in the presence of epinephrine. Plasmid-mediated expression of *qseBC* suppressed bacterial growth, complicating attempts to *trans*-complement mutant phenotypes. Our data support a role for QseBC in motility, biofilm formation and net intracellular survival of *B*. *pseudomallei*, but indicate that it is not essential for epinephrine-induced motility *per se*.

## Introduction

*Burkholderia pseudomallei* is a motile Gram-negative pathogen that causes melioidosis, a severe invasive human disease endemic in Southeast Asia and Northern Australia [1]. It has been estimated that 165,000 cases of meliodiosis occur globally each year, causing 89,000 deaths [2]. *B*. *pseudomallei* is prevalent in soil and water in endemic areas, and inhalation and injury are believed to be important routes of infection. Clinical presentations vary, but frequently include acute pneumonia, septicaemia and abscess formation [3]. No vaccine is available for melioidosis and antibiotic treatment is hindered by intrinsic and transmissible drug resistances. Targeted and genome-wide mutagenesis has identified numerous virulence factors of *B*. *pseudomallei* [4, 5]. Among the key factors required for invasion, escape from endosomes and intracellular net replication of *B*. *pseudomallei* is a Type III secretion system encoded by the *bsa* locus [6].

Two-component sensory systems play a key role in regulation of virulence genes in Gram-negative bacteria, including pathogenic *Burkholderia* [7]. These comprise a histidine kinase which senses an environmental cue, and on receipt of this, phosphorylates a cognate transcriptional regulator. This typically activates the regulator, altering the transcription of genes under its control. Among the two-component systems so far implicated in the virulence of *B*. *pseudomallei* in murine models are the BprRS system [8], BPSL0127-BPSL0128 system [9] and the VirAG system that regulates a virulence-associated Type VI secretion system in response to cytosolic glutathione [10]. Here, we identified homologues of the QseBC system in *B*. *pseudomallei*, which has been implicated in virulence in diverse Gram-negative bacterial pathogens.

The QseBC system (quorum sensing *E*. *coli* regulators B and C) was first described as a regulator of motility and flagellar gene expression in enterohaemorrhagic *Escherichia coli* O157:H7 in response to quorum sensing [11]. It was later reported that in addition to sensing the bacterial autoinducer AI-3, QseC senses the host stress-related catecholamine hormones epinephrine and norepinephrine [12]. In addition to activating motility, epinephrine and norepinephrine were reported to enhance expression of virulence-associated genes, including a Type III secretion system associated with intestinal colonisation [12, 13]. Epinephrine and norepinephrine have further been reported to stimulate biofilm formation in *E*. *coli* in a manner that requires QseC [14] and the QseBC system has been associated with biofilm formation by mastitis-associated *E*. *coli* [15].

QseBC homologues have been identified in diverse Gram-negative pathogens, but predicting the role of these in virulence is complicated by significant disparities in mutant phenotypes observed by different laboratories. For example, in contrast to the findings of Sperandio et al. [11], Sharma et al. [16] found no impact of a *qseBC* deletion on motility of *E*. *coli* O157:H7 or transcription of *fliC*, and the mutant colonized the bovine intestines at a higher level than the parent strain in co-infection studies. Independently, Hamed et al. [17] reported that a *qseC* deletion did not significantly alter motility of *E*. *coli* O157:H7 in the presence or absence of epinephrine or norepinephrine. Similarly, while QseC has been reported to be required for norepinephrine-induced motility in *Salmonella enterica* serovar Typhimurium [18] and for epinephrine-dependent regulation of Type III secretion systems, intra-macrophage survival and virulence in this organism [19, 20], Pullinger et al. [21] did not observe such impacts. The latter authors found no defect in motility or transcription of Type III secretion in a *S*. Typhimurium *qseC* mutant, and while norepinephrine induced bacterial growth and augmented inflammatory and secretory responses in a bovine ligated ileal loop model, this occurred at a similar level without *qseC*. Consistent with these studies, Hamed et al. [17] reported that motility of an *S*. Typhimurium *qseC* mutant was comparable to the wild-type in the presence or absence of epinephrine, norepinephrine or dopamine.

We previously reported that epinephrine activates motility and flagellar gene expression in *B*. *pseudomallei* [22]. Given literature on the role of QseBC in this phenotype in other pathogenic bacteria, we identified homologues of QseBC, constructed an unmarked *qseBC* deletion mutant and analysed the impact of this on epinephrine-induced motility, biofilm formation and intracellular net replication.

## Materials and methods

### Bacterial strains and culture conditions

We used the prototype genome-sequenced *B*. *pseudomallei* strain K96243 [23], which has been extensively studied in our laboratories. K96243 wild-type, Δ*qseBC* mutant and Δ*qseBC trans*-complemented strains were grown in Luria-Bertani broth or agar (LB; Titan Biotech Ltd., Delhi, India) at 37°C for 24–48 h. A previously described *bsaZ* mutant strain was used as a control in intracellular survival assays [6]. Chloramphenicol (40 μg/mL), kanamycin (1 mg/mL) or zeocin (2 mg/mL) were used for selection where required. *E*. *coli* K-12 strains DH5α and RHO3 [24] were cultured in LB agar or broth at 37°C for 18–24 h. To support growth of *E*. *coli* RHO3, 2,6 diaminopimelic acid (DAP; Sigma, St. Louis, MO) was added to the culture medium. Chloramphenicol (30 μg/mL) and kanamycin (35 μg/mL) were added when needed for *E*. *coli*. Strain genotypes and sources are described in S1 Table.

### Construction of a *B*. *pseudomallei* F044*qseBC* mutant

An unmarked deletion mutant of *B*. *pseudomallei* strain K96423 lacking *qseBC* was constructed essentially as described [24]. We used PCR to amplify a region 201-bp upstream of the gene encoding putative the QseB response regulator (*bpsl0806*) and 393-bp downstream of the gene encoding putative the QseC sensor kinase (*bpsl0807*) and then joined these by overlap extension PCR using primers described in S2 Table as previously described [24]. The resulting PCR product was cloned into the pGEM-T easy vector (Promega, USA) and the insert was then subcloned into the positive-selection suicide replicon pEXKm5 [24] by digestion with *Eco*RI, yielding plasmid pEX*qseBC*. *E*. *coli* RHO3 harboring pEX*qseBC* was used to introduce the plasmid into *B*. *pseudomallei* K96243 by conjugation. Merodiploids resulting from homologous recombination were selected by plating on LB agar containing 1 mg/mL kanamycin. To select for double recombinants in which the suicide replicon was lost, homing nuclease I-*Sce*-I-mediated gene replacement was performed. The pBADSce vector [24] was introduced into a merodiploid strain by electroporation. Clones were selected by plating on LB agar containing 2 mg/mL zeocin (Invivogen, California, USA) with 0.5% (w/v) L-arabinose and then incubated at 30°C for 36 h. After incubation, putative double recombinants were patched on LB with or without 1 mg/mL kanamycin. A kanamycin sensitive colony was grown in LB at 42°C to select for loss of the pBADSce vector. A double recombinant lacking *qseBC* mutant was initially identified by PCR using primers flanking the *qseBC* genes (S2 Table).

### Whole-genome sequencing and bioinformatic analysis

Genomic DNA of the *B*. *pseudomallei* Δ*qseBC* mutant was extracted using a Genomic DNA mini kit (Geneaid Biotech, Taiwan). Whole-genome sequencing was performed by Illumina Miseq sequencing (San Diego, CA, USA) at the ‘Omics Sciences and Bioinformatic Center at Chulalongkorn University, Bangkok, Thailand. Quality of the sequence reads was checked using FASTQC software [25]. The average coverage of sequence reads was 100X. Sequence reads were aligned to the K96423 reference genome [23] (accession number NC_006350 and NC_006351) using BWA mem v0.7.12. Alignments to the K96243 reference genome were also used to identify single nucleotide polymorphisms (SNPs), insertions or deletions using Pilon (v1.22) [26]. Integrative Genomics Viewer was used to visualise the site of the deletion on chromosome 1. The nucleotide sequence of the K96243 Δ*qseBC* strain has been deposited in GenBank (accession number JAJOLO000000000). Raw sequence reads were deposited in the Short Read Archive (accession number PRJNA785800).

Homology of *qseBC* genes in *B*. *pseudomallei* relative to those in *E*. *coli* and other *Burkholderia* species was assessed by the UniProt Tool, Ident and Sim software and Simple Modular Architecture Research tool. Accession numbers of the query and target sequences used are listed in the corresponding figure legend.

### Cloning of the *B*. *pseudomallei qseBC* genes for *trans*-complementation

The *qseBC* genes were amplified by with primers Com_BPSL0806-F and Com_BPSL0807-R (S2 Table), including the predicted ribosome-binding sequence 5’ of *qseB* to enable translation. The amplicon was digested with *Kpn*I and *Xba*I restriction enzymes and cloned in the pBBR1MCS-1 broad host-range vector [27]. The sequence of the insert was confirmed to be as expected by Sanger sequencing (S1 Fig). The resulting plasmid (pBBR*qseBC*) was introduced into the *B*. *pseudomallei* Δ*qseBC* mutant by conjugation from *E*. *coli* strain RHO3 with selection for chloramphenicol resistance. Transcription of the *qseBC* genes in wild-type, mutant and *trans*-complemented strains was analysed by reverse-transcriptase PCR as described below.

### Motility assays

Swimming and swarming motility assays were performed by measuring the diameter of motility zone on motility plates according to Déziel et al. [28] with some modifications. *B*. *pseudomallei* strains were grown overnight and adjusted to an optical density at 600nm (OD_600_) of 0.5. Then, 3 μl of the adjusted suspension was carefully spotted onto 0.3% (w/v) or 0.5% agar plates to respectively investigate swimming and swarming, that contained yeast extract (3 g/L; Titan Biotech Ltd., Delhi, India), tryptone (5 g/L; Hardy Diagnostics, Ohio, USA), and glucose (5 g/L; Ajax Finechem, Australia), with or without 50 μM epinephrine hydrochloride (Sigma, St. Louis, MO). After inoculation, plates were incubated at 37°C for 18 h. The diameter of the zone of bacterial swimming or swarming motility was measured on the following day. Results represented the mean diameter of the motility zones. The experiment was performed with three technical replicates each performed on three separate occasions.

### Biofilm formation assay

Biofilm formation was assayed essentially as described [29] with the following modifications. Overnight bacterial cultures were adjusted to a concentration of 1x10^8^ colony-forming units (CFU)/mL and then inoculated into 96-well plates with or without 50 μM epinephrine. The plates were then incubated statically at 37°C for 72 h. After incubation, non-adherent cells were removed by washing twice in phosphate-buffered saline (PBS; Sigma, St. Louis, MO). The biofilm was then fixed using 99% (v/v) methanol for 15 minutes and stained with 0.1% (w/v) crystal violet (Merck, New Jersey, USA) for 15 minutes. Finally, crystal violet was solubilized using 33% (v/v) glacial acetic acid (VWR international, Pennsylvania, USA). Optical density of the solubilized crystal violet suspension was then measured at 550 nm using a microplate reader (TECAN, Switzerland). Assays were performed with eight technical replicates on three separate occasions.

### RNA extraction and real-time reverse transcriptase-PCR

*B*. *pseudomallei* strains were subcultured in 10 mL of LB broth with or without 50 μM epinephrine and grown at 37°C. Logarithmic phase cells were harvested at OD_600_ of 0.5 and total RNA was extracted using a Total RNA-mini kit (Geneaid Biotech, Taipei, TW). Total RNA was treated with RNase-free DNase I (Thermo Scientific, MA, USA). We confirmed the absence of DNA by PCR using primers specific to 16S rRNA genes (S2 Table). RNA was then reverse transcribed to cDNA by using the SuperScript III First-Strand Synthesis System (Invitrogen, Carlsbad, CA), according to the manufacturers instructions.

Analysis of the transcription of the flagellin gene (*fliC*) and *qseBC* gene was performed by real-time PCR using the cDNA samples obtained above and gene-specific primers (S2 Table) using a LightCycler 480 instrument (Roche diagnostics, Penzburgh, Germany). Relative gene expression was calculated using the 2^-ΔΔCt^ method [30] with normalization relative to 16S ribosomal RNA. To detect *qseBC* transcripts in wild-type, mutant or *trans*-complemented strains, conventional RT-PCR was performed with gene-specific primers (S2 Table) and amplicons were resolved by agarose gel electrophoresis.

### Analysis of bacterial growth kinetics

*B*. *pseudomallei* K96243 wild-type, Δ*qseBC* mutant and Δ*qseBC/*pBBR*qseBC trans*-complemented strains were grown overnight and adjusted to reach OD_600_ value of 0.17. Then, 300 μl of the adjusted bacterial suspension was then added to 15 mL of fresh LB broth. At 0, 2, 4, 8, 12 and 24 h, OD_600_ measurements were taken to generate bacterial growth curves. At these same intervals, ten-fold serial dilutions of samples of the cultures were prepared and spotted onto LB agar plates. The plates were incubated at 37°C for 24–48 h and then viable bacteria were enumerated and expressed as CFU/mL. Experiments were performed with three technical replicates on three separate occasions.

### Intracellular survival assay

J774A.1 murine macrophage cells were used to analyse net intracellular survival of the strains. The cells were maintained in Dulbecco’s modified Eagle’s medium (DMEM; Gibco, Invitrogen, Carlsbad, CA) supplemented with 10% (v/v) heat-inactivated foetal bovine serum (HyClone, South Logan, UT) at 37°C in a humidified 5% CO_2_ atmosphere. J774A.1 cells were seeded at 2x10^5^ per well in 24-well plates. They were then inoculated with *B*. *pseudomallei* wild-type or mutant strains at a multiplicity of infection of 0.1. Plates were centrifuged at 13x *g* for 5 minutes to drive bacterial into contact with the macrophages and obviate any effects on flagella-mediated motility. After 1 h post-inoculation, the infected cells were overlaid with DMEM supplemented with 10% (v/v) heat-inactivated foetal bovine serum containing 250 μg/mL kanamycin to eliminate extracellular bacteria. At 2, 4, 6, 8 and 24 h post-infection, the infected cells were washed with pre-warmed PBS and lysed with 0.1% (v/v) Triton X-100 (Sigma, St. Louis, MO) in PBS for 5 minutes. The viable intracellular bacteria released were enumerated following plating of serial ten-fold dilutions to LB agar plates and incubation for 24–28 h.

### Statistical analysis

Statistical analysis was performed by using a student’s *t*-test using GraphPad Prism8 software. Differences were considered significant at *P* values of ≤ 0.05.

## Results and discussion

### *B*. *pseudomallei* BPSL0806 and BPSL0807 are homologues of *E*. *coli* QseB and QseC, respectively

BLAST analysis of the complete genome sequence of *B*. *pseudomallei* K96243 [23] using *E*. *coli* O157:H7 *qseB* and *qseC* genes as query sequences identified *bpsl0806* and *bpsl0807* on chromosome 1 as the closest homologues, respectively. BPSL0806 is predicted to be the response regulator and to be a 220 amino acid protein that is 50.91% identical and 66.36% similar to *E*. *coli* O157:H7 QseB over 220 amino acids. BPSL0807 is predicted to be the sensor kinase and 438 amino acids in length, sharing 28.88% identity and 48.49% similarity with *E*. *coli* O157:H7 QseC over 449 amino acids. We analysed the conservation of 8 residues that have been reported to be conserved in the predicted periplasmic domains of QseC homologues from 12 bacteria [31]. This revealed that 6 of these amino residues were shared in *B*. *pseudomallei* QseC (S2 Fig). Protein sequences of *B*. *pseudomallei* QseBC shared 99–100% identity with the corresponding proteins *Burkholderia mallei* ATCC23344 and 90–97% identity with those from *Burkholderia thailandensis* E264 (S3 Table). Across representatives of the *B*. *cepacia* complex and phytopathogenic *Burkholderia*, QseB exhibited at least 83% identity and 85% similarity with the *B*. *pseudomallei* protein, and QseC was at least 80% identical and 85% similar (S3 Table).

Analysis of the transcriptome of *B*. *pseudomallei* strain K96243 under 82 different conditions using a tiling whole-genome microarray indicated that the *bpsl0806-0807* are transcribed [32]. In particular, the authors demonstrated that *bpsl0806* is up-regulated within 1 h of nutrient-deprivation. We confirmed that a transcript spanning the *qseBC* genes could be detected in K96243 during growth in LB medium. This finding indicated that *qseB* and *qseC* are co-transcribed as an operon. The abundance of the transcript was unchanged in the presence of 50 μM epinephrine (S3 Fig).

### Generation of *B*. *pseudomallei qseBC* mutant and *trans*-complemented strains

An unmarked Δ*qseBC* deletion mutant was constructed using a suicide replicon containing a fusion of amplicons spanning 201-bp 5’ of *bpsl0806* and 393-bp 3’ of *bpsl0807*, as described in Materials and Methods. A putative double recombinant was confirmed to harbor the *qseBC* deletion by PCR with flanking primers (S4 Fig) and was then sequenced using the Illimina MiSeq platform. Sequence reads were aligned to the original K96243 reference genome, which confirmed the location of the expected deletion on chromosome 1 (S5 Fig). Importantly, we observed no unwanted rearrangements of the genome, including proximal to the deletion site, as has been reported following the use of suicide vectors for allelic exchange [33]. Sequence analysis identified a number of candidate polymorphisms and small insertions or deletions (indels) relative to the original published sequence for K96243 (S4 Table). Specifically, 44 SNPs and 9 indels were identified across chromosomes 1 and 2. The vast majority of these SNPs and indels have been previously reported following resequencing of four K96243 stocks from different laboratories [34, 35]. They affect a total of 10 genes while 12 are in intergenic regions. Just two SNPs associated with missense mutations in *bpsl1036* (a hypothetical gene with homology to *ompR*) and *bpss1755* (proximal to the C-terminus of the RpoD sigma factor), and a single nucleotide insertion predicted to cause a frameshift mutation in *bpss1194*, were unique relative to K96243 strains sequenced to date (S4 Table). A large number of other SNPs were detected in *bpss1194* that are shared with other isolates. The extent to which polymorphisms could be due to sequencing errors in our analysis or with the reference strain is unclear.

As we cannot preclude the possibility that minor variants detected in the K96243 Δ*qseBC* genome sequence may contribute to its phenotypes, we cloned the *qseBC* genes under the control of an inducible promoter in the broad-host range vector pBBR1MCS-1 (S6A Fig). This was introduced into the mutant strain and we confirmed by reverse-transcriptase PCR that the *qseBC* transcript was present in the wild-type and *trans*-complemented strains, but absent in the Δ*qseBC* mutant (S6B Fig). We analysed the growth kinetics of the wild-type K96243 strain, Δ*qseBC* mutant and Δ*qseBC/*pBBR*qseBC trans*-complemented strain in LB broth by measuring both optical density and viable bacteria over time. Growth of the wild-type and Δ*qseBC* mutant strains was almost identical, indicating that polymorphisms detected by genome sequencing have not grossly affected bacterial fitness. However, we observed that growth of the Δ*qseBC/*pBBR*qseBC* strain was significantly suppressed at all the time intervals studied (S6C and S6D Fig). This is consistent with the phenotype of an *E*. *coli* O157:H7 *qseBC* mutant upon plasmid-mediated expression of the *qseBC* genes [16]. It is possible that this may be explained by recent data indicating that QseBC controls the timing of initiation of chromosomal replication in *E*. *coli*, likely by regulating expression of *dnaA* [36].

### The *B*. *pseudomallei qseBC* genes influence motility and *fliC* transcription

**Fig 1:**
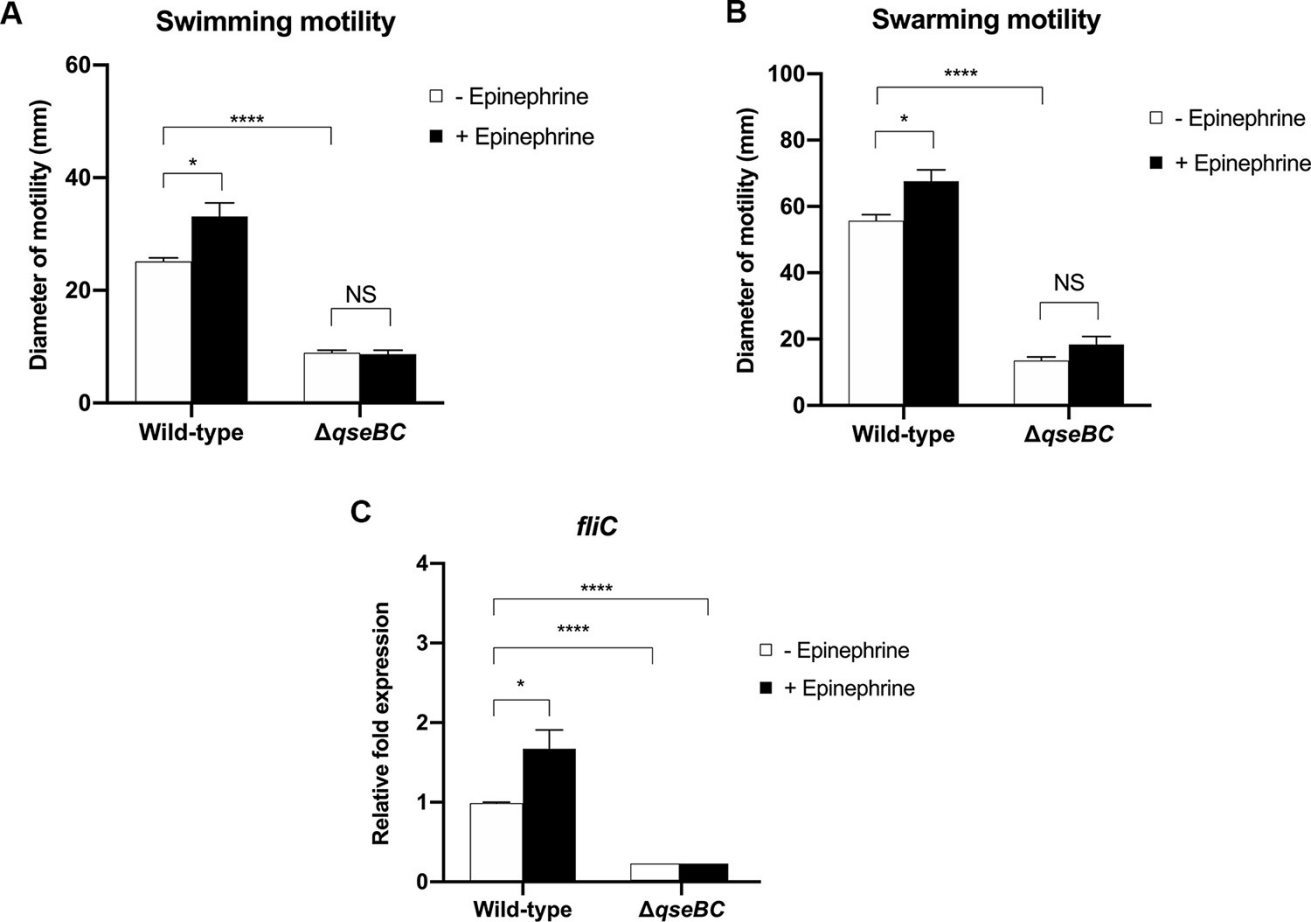
QseBC homologues are required for *B*. *pseudomallei* motility. *B*. *pseudomallei* wild-type K96243 and Δ*qseBC* mutant were grown on motility plates in the presence or absence 50 μM epinephrine. Motility zones after 18 h of incubation were determined. (A) 0.3% (w/v) nutrient agar was used to assess for swimming motility. (B) Swarming motility was assayed using 0.5% (w/v) nutrient agar. (C) Transcription of *fliC* gene was investigated by real-time reverse transcriptase PCR using *B*. *pseudomallei* wild-type K96243 and Δ*qseBC* mutant cultured to exponential phase in LB medium in the presence or absence 50 μM epinephrine. Three independent experiments were performed. Error bars represent standard errors of the means following students *t*-test. Asterisks indicate significant differences (*P* ≤ 0.05, *t*-test).

Motility of the *B*. *pseudomallei* K96243 wild-type and Δ*qseBC* mutant strains was analysed on plates containing 0.3% (w/v) or 0.5% (w/v) agar to measure swimming and swarming motility, respectively. In soft agar (0.3% w/v), bacterial cells swim through the relatively fluid agar suspension while bacteria swarm over the surface when agar is present a 0.5% (w/v) [37]. We observed a significant reduction in both swimming motility (Fig 1A; c. 2.8-fold *P* <0.0001) and swarming motility (Fig 1B; c. 4.1-fold *P* = <0.0001) between the wild-type strain and Δ*qseBC* mutant. Moreover, we observed a reduction in transcription of the *fliC* gene encoding flagellin in the Δ*qseBC* mutant (Fig 1C; c. 132.1-fold *P* = <0.0001). We were unable to rescue the motility defect of the mutant strain by introduction of pBBR*qseBC*, likely as a consequence of markedly suppressed growth (S6C and S6D Fig). We also attempted to repair the *qseBC* mutant by reintroduction of the intact *qseBC* genes using a positive-selection suicide replicon, but were unsuccessful.

Consistent with our previous research [22], 50 μM epinephrine significantly enhanced swimming motility Fig 1A; c. 1.3 fold *P* = 0.0311), swarming motility of *B*. *pseudomallei* K96243 (Fig 1B; c. 1.2-fold *P* = 0.0385) and transcription of *fliC* (Fig 1C; c. 1.7- fold *P* = 0.0464). Swimming motility, swarming motility and *fliC* transcription in the Δ*qseBC* mutant were near-identical in the presence and absence of 50 μM epinephrine. Thus, QseBC is required for full motility but epinephrine does not have to be present for it to regulate it. While QseBC have been reported to act as adrenergic sensors controlling motility in *E*. *coli* O157:H7 [12] and *S*. Typhimurium [18], other laboratories have found no defects in motility for *qseBC* mutants of these pathogens [16, 17, 21]. The extent to which this reflects differences in the strains, types of mutation and assay conditions remains unclear.

### The *B*. *pseudomallei qseBC* genes influence biofilm production

**Fig 2:**
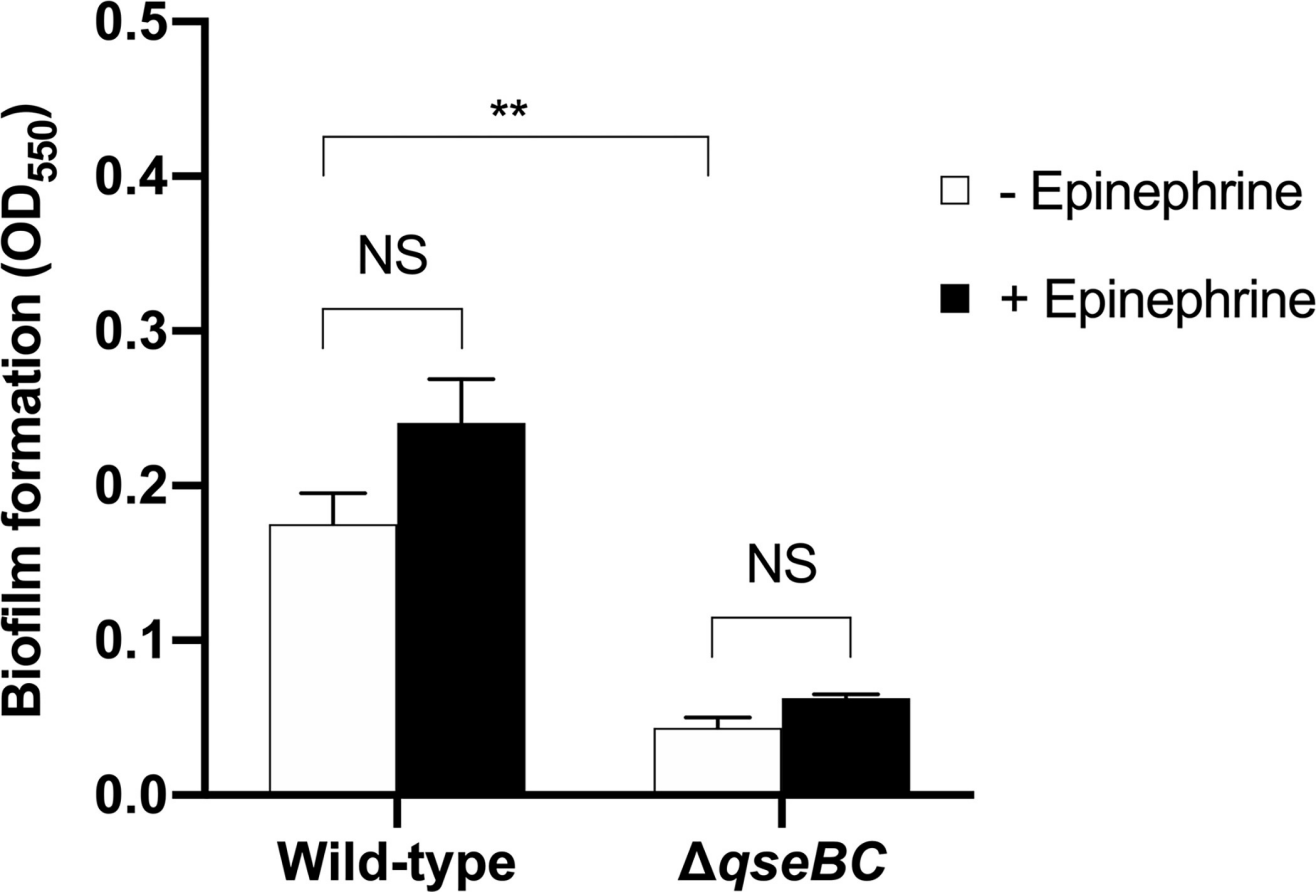
Effect of *qseBC* mutation and epinephrine on biofilm formation by *B*. *pseudomallei*. Biofilm formations by *B*. *pseudomallei* wild-type K96243 and its **Δ***qseBC* mutant was measured after incubation in LB broth supplemented with or without 50 μM epinephrine for 72 h. Three independent experiments were performed. Error bars represent standard errors of the means. Asterisks indicate significant differences following students *t*-test (*P* ≤ 0.05, *t*-test).

Biofilms can contribute to bacterial pathogenesis and aid resistance to antibiotics and host defences [38]. We observed that biofilm formation by strain K96243 was significantly reduced by the Δ*qseBC* mutation (Fig 2; c. 4-fold *P* = 0.0034). It is plausible that this is a consequence of the impact of the Δ*qseBC* mutation on flagella expression, as a genome-wide screen of transposon mutants for defects in biofilm formation identified mutations in multiple flagellum-related genes [39]. In the presence of 50 μM epinephrine, a slight but non-significant increase in biofilm formation was detected for both the wild-type and Δ*qseBC* mutant strains (Fig 2). The impact of Δ*qseBC* mutation on biofilm formation by *B*. *pseudomallei* is consistent with reports using *qseC* mutants of *Haemophilus parasuis* [40]. *Aggregatibacter actinomycetemcomitans* [41], *E*. *coli* strains [14, 15], and a *qseB* mutant of *Salmonella* Typhi [42]. We consider it unlikely that the missense mutation detected in *bpsl1036* accounts for the phenotype of the Δ*qseBC* mutant, as deletion of this gene in *B*. *pseudomallei* has been reported to increase biofilm formation [43].

### The *B*. *pseudomallei qseBC* genes influence net intracellular survival in macrophages

**Fig 3:**
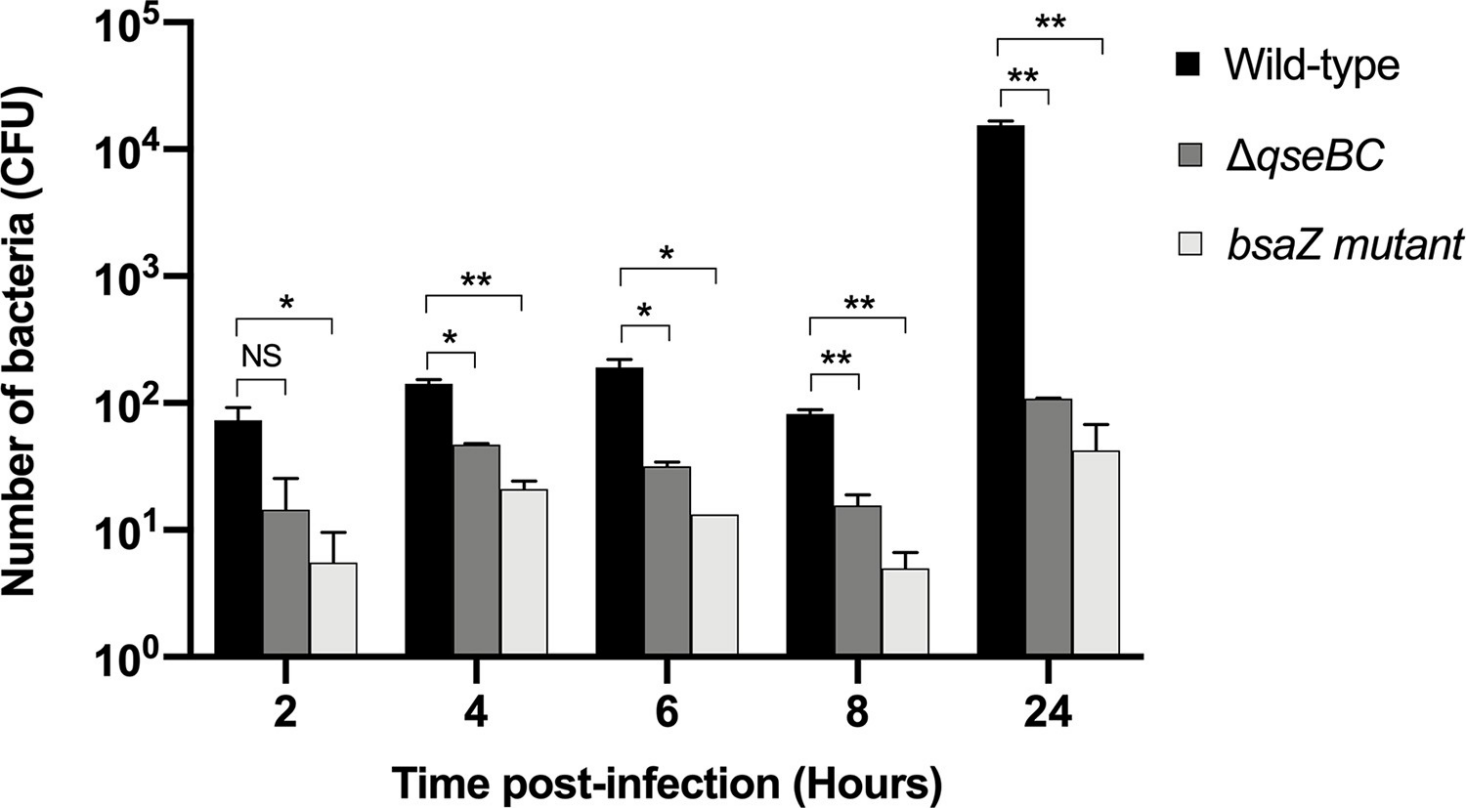
Effect of *qseBC* mutation on the intracellular survival of *B*. *pseudomallei* in J774A.1 macrophage-like cells. Murine J774A.1 macrophages were infected with either *B*. *pseudomallei* wild-type K96243 or its Δ*qseBC* mutant at an MOI 0.1. At 2, 4, 6, 8 and 24 h post-infection, infected cells were lysed with 0.1% Triton X-100 and the numbers of viable intracellular bacteria were enumerated. A *B*. *pseudomallei bsaZ* mutant was included as positive control. Two independent experiments were performed. Error bars represent standard errors of the means. Asterisks indicate significant differences following students *t*-test (*P* ≤ 0.05, *t*-test) compared with the *B*. *pseudomallei* wild-type strain.

*B*. *pseudomallei* is a facultative intracellular pathogen and factors that influence its survival in macrophages are known to be important for virulence in murine models of melioidosis [4, 44]. We studied uptake and net intracellular survival of *B*. *pseudomallei* K96243 and the Δ*qseBC* mutant in J774A.1 murine macrophage-like cells over time. A *bsaZ* mutant known to be attenuated in this model was included as a control [6]. After inoculation, bacteria were driven into contact with the macrophages by centrifugation with the aim of avoiding indirect effects due to impaired motility. At 2 h post-infection (1 h to allow uptake and 1 h for kanamycin to kill extracellular bacteria), the number of intracellular (kanamycin-protected) Δ*qseBC* mutant bacteria was not significantly different than the wild-type implying comparable uptake (Fig 3). However, at 4, 6, 8 and 24 h post-infection the numbers of intracellular Δ*qseBC* mutant were significantly reduced compared to the wild-type strain (*P* = 0.0120, *P* = 0.0306, *P* = 0.0099 and *P* = 0.0060, respectively). A significant difference was also detected for the mutant lacking the function of the Bsa Type III secretion system as expected [6]. Sequence analysis of the Δ*qseBC* mutant revealed a missense mutation resulting in a predicted a single amino acid substitution in BapA, an effector protein injected by the Bsa system (S4 Table). However, it is considered unlikely that this affects the phenotype of the Δ*qseBC* mutant as a *bapA* mutant exhibited no defect in invasion or net survival in epithelial or macrophage cell lines [45].

A role for the QseBC system in intracellular life of *B*. *pseudomallei* is consistent with the phenotype in J774 macrophages of *qseC* mutants of *Edwardsiella tarda* [46] and *S*. Typhimurium [20]. Introduction of pBBR*qseBC* into the Δ*qseBC* mutant strain did not rescue the ability of the mutant strain to survive in J774A.1 cells to wild-type levels likely owing to suppression of growth as evident from S6C and S6D Fig.

## Conclusions

We identified homologues of the *E*. *coli* QseBC proteins in *B*. *pseudomallei* (BPSL0806 and BPSL0807). A Δ*qseBC* non-polar deletion mutant was constructed and verified to be near-identical at the whole genome sequence level to sequenced K96243 strains. Consistent with observations in other pathogenic bacteria, deletion of *B*. *pseudomallei qseBC* impaired motility, *fliC* transcription, biofilm formation and intra-macrophage survival. While QseBC controls similar phenotypes in *B*. *pseudomallei* to those described in other bacteria, epinephrine does not need to be present for it to do so.

## Supporting information

S1 Fig Confirmation of the sequence of the *qseBC* genes inserted in pBBR1MCS-1 by Sanger sequencing. Nucleotide sequences were aligned using Bioedit version 7.2.5. The *B*. *pseudomallei* K96243 *qseBC* nucleotide sequence (accession number: NC_006350.1) was compared with the sequence obtained for the insert in pBBR*qseBC*. The predicted ribosome-binding site sequence is highlighted in yellow.
(PDF)

S2 Fig Multiple protein sequence alignments of *B*. *pseudomallei* QseB and QseC relative to homologous proteins from other *Burkholderia* species and *E*. *coli* O157:H7. QseB and QseC amino acid sequences from *B*. *pseudomallei* K96243 (UniProt accession numbers: Q63WT5, Q63WT4) was compared with those from *B*. *mallei* ATCC 23344 (UniProt accession numbers: A0A0H2WJH0, A0A0H2WIM0), *B*. *thailandensis* E264 (UniProt accession numbers: Q2T0S0, Q2T0R9), *B*. *cepacia* ATCC25416 (UniProt accession numbers: A0A806UVQ1, A0A806V178), *B*. *cenocepacia* ATCCJ2315 (UniProt accession numbers: B4EA15, B4EA14), *B*. *multivorans* ATCC17616 (UniProt accession numbers: A0A0H3KLT5, A0A0H3KH15), *B*. *gladioli* (UniProt accession numbers: A0A095FGQ9, A0A095FGJ4), *B*. *dolosa* AU0158 (UniProt accession numbers: A2W7S9, A2W7T0), *B*. *glumae* BGR1 (UniProt accession numbers: C5ACR6, C5ACR5) and *E*. *coli* O157:H7 (UniProt accession numbers: Q8XBS3, Q8X524). Residues in the periplasmic domain of *E*. *coli* O157:H7 QseC that are conserved across QseC homologues from diverse bacteria are highlighted in yellow. The symbols demonstrate the scale of conservation, whereby asterisk (*) indicates a fully conserved residue, colon (:) indicates conservation between groups of strongly similar properties and period (.) indicates conservation between groups of weakly similar properties. The functional domains were predicted using a Simple Modular Architecture Research Tool or SMART (http://smart.embl.de). The functional regions were highlighted in different colors (green, receiver domain; blue, transcriptional regulatory domain; grey, transmembrane region; orange, HAMP domain; pink, histidine kinase domain; brown, histidine kinase-like ATPase).
(PDF)

S3 Fig Transcription of the *B*. *pseudomallei qseBC* genes is not affected by epinephrine. *B*. *pseudomallei* wild-type K96243 was grown in LB medium with or without 50μM epinephrine. The transcription of *qseBC* was analysed by real-time reverse transcriptase PCR. Three independent experiments were performed. Error bars represent the standard error of the mean. NS indicates no significant difference following a student’s *t*-test.
(TIF)

S4 Fig Verification of *B*. *pseudomallei* Δ*qseBC* mutation. PCR analysis of *B*. *pseudomallei* wild-type K96243 compared with the Δ*qseBC* mutant using primers BPSL0806-F and BPSL0807-R. The amplicon predicted to be generated from *B*. *pseudomallei* wild-type is 2639-bp (lane 1) whereas for the Δ*qseBC* mutant it is 574-bp (lane 2). Lane M and N represent 1 kb DNA ladder and negative control, respectively.
(TIFF)

S5 Fig Whole genome alignment between *B*. *pseudomallei* K96243 and Δ*qseBC* mutant using Integrative Genomics Viewer (IGV). The sequence genomes of K96243 and Δ*qseBC* mutant were compared with each other in chromosome 1. Deletions between *bpsl0806* and *bpsl0807* in *B*. *pseudomallei* Δ*qseBC* mutant were shown. There were no effects on adjacent genes.
(TIFF)

S6 Fig Construction of a *B*. *pseudomallei qseBC*-complemented strain. (A) Schematic representation of generation of plasmid pBBR*qseBC*. (B) RT-PCR analysis of *qseBC* expression using *B*. *pseudomallei* wild-type K96243 cDNA (lane 2), Δ*qseBC* mutant cDNA (lane 3) and Δ*qseBC*/pBBR*qseBC* cDNA (lane 4). Positive control using gDNA wild-type is shown in lane 1. Lanes M and N represent 100-bp DNA ladder and negative control, respectively. *B*. *pseudomallei* wild-type K96243, Δ*qseBC* mutant and Δ*qseBC*/pBBR*qseBC* complemented strains were grown in LB medium and bacterial cells were collected at 2, 4, 8, 12 and 24 h. for OD measurement (C) and colony count (D). Data represents the mean of triplicate determinations.
(TIF)

S1 Table Bacterial strains and plasmids used in this study. (PDF)

S2 Table List of primers used in this study. (PDF)

S3 Table Identity and similarity of homologues of QseB and QseC across *Burkholderia* species. (PDF)

S4 Table Analysis of nucleotide variation in the *B*. *pseudomallei* Δ*qseBC* mutant genome compared to the original reference K96243 genome and resequencing data for different laboratory stocks. (XLSX)
