# Supplementary material for: Analysis of the role of the QseBC two-component sensory system in epinephrine-induced motility and intracellular replication of Burkholderia pseudomallei
Source: PLoS One. 2023 Feb 23;18(2):e0282098. doi: 10.1371/journal.pone.0282098 (PMC9949665; doi:10.1371/journal.pone.0282098)
Supplement: S1 Fig — Nucleotide sequences were aligned using Bioedit version 7.2.5. The B. pseudomallei K96243 qseBC nucleotide sequence (accession number: NC_006350.1) was compared with the sequence obtained for the insert in pBBRqseBC. The predicted ribosome-binding site sequence is highlighted in yellow. (PDF) [file pone.0282098.s001.pdf]

S1 Fig. Confirmation of the sequence of the *qseBC* genes inserted in pBBR1MCS-1 by Sanger sequencing. Nucleotide sequences were aligned using Bioedit version 7.2.5. The *B. pseudomallei* K96243 *qseBC* nucleotide sequence (accession number: NC_006350.1) was compared with the sequence obtained for the insert in pBBR*qseBC*. The predicted ribosome-binding site sequence is highlighted in yellow.

```
                           10        20        30        40        50        60        70        80        90       100       110       120
B.pseudomallei K96243  --------------ATGCGGATATTGCTGGTTGAAGACGATCGGATGATCGCCGAAGGCGTGCGCAAGGCGCTGAAGGCCGACGGCTGCGCGGTCGACTGGGTGCAGGACGGCGACGCGG
                                     ||||||||||||||||||||||||||||||||||||||||||||||||||||||||||||||||||||||||||||||||||||||||||||||||||||||||||
pBBRqseBC              AGGAGGTTGCCATCATGCGGATATTGCTGGTTGAAGACGATCGGATGATCGCCGAAGGCGTGCGCAAGGCGCTGAAGGCCGACGGCTGCGCGGTCGACTGGGTGCAGGACGGCGACGCGG

                          130       140       150       160       170       180       190       200       210       220       230       240
B.pseudomallei K96243  CGCTCACCGCGCTCGGCGGCGAGGCGTACGATCTGCTGCTGCTCGATCTCGGGCTGCCCAAGCGCGACGGCATCGACGTGCTGCGCACGCTGCGCGCGCGCGGGCTCGCGCTGCCGGTGC
                       ||||||||||||||||||||||||||||||||||||||||||||||||||||||||||||||||||||||||||||||||||||||||||||||||||||||||||||||||||||||||
pBBRqseBC              CGCTCACCGCGCTCGGCGGCGAGGCGTACGATCTGCTGCTGCTCGATCTCGGGCTGCCCAAGCGCGACGGCATCGACGTGCTGCGCACGCTGCGCGCGCGCGGGCTCGCGCTGCCGGTGC

                          250       260       270       280       290       300       310       320       330       340       350       360
B.pseudomallei K96243  TGATCCTCACCGCGCGCGATGCGGTCGCCGATCGCGTGAAGGGCCTCGACGCGGGCGCCGACGACTATCTCGTCAAGCCGTTCGATCTCGACGAGCTCGCCGCGCGGATGCGCGCGCTGA
                       ||||||||||||||||||||||||||||||||||||||||||||||||||||||||||||||||||||||||||||||||||||||||||||||||||||||||||||||||||||||||
pBBRqseBC              TGATCCTCACCGCGCGCGATGCGGTCGCCGATCGCGTGAAGGGCCTCGACGCGGGCGCCGACGACTATCTCGTCAAGCCGTTCGATCTCGACGAGCTCGCCGCGCGGATGCGCGCGCTGA

                          370       380       390       400       410       420       430       440       450       460       470       480
B.pseudomallei K96243  TCCGCCGCCAGTCGGGGCGCAGCGAATCGATGATCCGGCACGGCGCGCTGACGCTCGATCCGGCGTCGCATCAGGTGACGCTCGACGGGGCGCCCGTCGCGCTGTCGGCGCGCGAGTTCG
                       ||||||||||||||||||||||||||||||||||||||||||||||||||||||||||||||||||||||||||||||||||||||||||||||||||||||||||||||||||||||||
pBBRqseBC              TCCGCCGCCAGTCGGGGCGCAGCGAATCGATGATCCGGCACGGCGCGCTGACGCTCGATCCGGCGTCGCATCAGGTGACGCTCGACGGGGCGCCCGTCGCGCTGTCGGCGCGCGAGTTCG

                          490       500       510       520       530       540       550       560       570       580       590       600
B.pseudomallei K96243  CGCTGCTCGAAGCGCTGCTCGCGCGTCCGGGCGCGGTGCTGTCGAAGAGCCAGCTCGAGGAGAAGATGTACGGCTGGGGCGAGGAGATCGGCAGCAACACCGTCGAGGTCTATATCCACG
                       ||||||||||||||||||||||||||||||||||||||||||||||||||||||||||||||||||||||||||||||||||||||||||||||||||||||||||||||||||||||||
pBBRqseBC              CGCTGCTCGAAGCGCTGCTCGCGCGTCCGGGCGCGGTGCTGTCGAAGAGCCAGCTCGAGGAGAAGATGTACGGCTGGGGCGAGGAGATCGGCAGCAACACCGTCGAGGTCTATATCCACG

                          610       620       630       640       650       660       670       680       690       700       710       720
B.pseudomallei K96243  CGCTGCGCAAGAAGCTCGGCTCGGACCTGATCCGCAACGTCCGCGGCCTCGGCTACATGGTCGTGAAGGAAGCGTAGCGCGTGAGGTCGATTCGCCATCAATTGCTGATCTGGCTGCTCG
                       ||||||||||||||||||||||||||||||||||||||||||||||||||||||||||||||||||||||||||||||||||||||||||||||||||||||||||||||||||||||||
pBBRqseBC              CGCTGCGCAAGAAGCTCGGCTCGGACCTGATCCGCAACGTCCGCGGCCTCGGCTACATGGTCGTGAAGGAAGCGTAGCGCGTGAGGTCGATTCGCCATCAATTGCTGATCTGGCTGCTCG

                          730       740       750       760       770       780       790       800       810       820       830       840
B.pseudomallei K96243  CGATCGTCGTGGCCGGCGTGGGGCTCGCCGGCTGGATGATCTACCGGCAGGCGCTTGCCGCCGCCAACGAGCTGTTCGACTATCAGCTGCAGCAGATCGCGGCCGCGCTGCCGTCCGAGC
                       ||||||||||||||||||||||||||||||||||||||||||||||||||||||||||||||||||||||||||||||||||||||||||||||||||||||||||||||||||||||||
pBBRqseBC              CGATCGTCGTGGCCGGCGTGGGGCTCGCCGGCTGGATGATCTACCGGCAGGCGCTTGCCGCCGCCAACGAGCTGTTCGACTATCAGCTGCAGCAGATCGCGGCCGCGCTGCCGTCCGAGC

                          850       860       870       880       890       900       910       920       930       940       950       960
B.pseudomallei K96243  CGTTCTCGCAGGTGCTCGGCTCGCAGACGAACGGCGACGAAGGGATCGTGATCCAGATCTGGAACCGCAACGGCGTGCTGATGTACTTCTCGCATCCGCGCGCGCCGATCGCCCCGCGCG
                       ||||||||||||||||||||||||||||||||||||||||||||||||||||||||||||||||||||||||||||||||||||||||||||||||||||||||||||||||||||||||
pBBRqseBC              CGTTCTCGCAGGTGCTCGGCTCGCAGACGAACGGCGACGAAGGGATCGTGATCCAGATCTGGAACCGCAACGGCGTGCTGATGTACTTCTCGCATCCGCGCGCGCCGATCGCCCCGCGCG

                          970       980       990      1000      1010      1020      1030      1040      1050      1060      1070      1080
B.pseudomallei K96243  CCGAGCTCGGCTTCTCGACCGAGCGCACCGAGCGCGGCGCGTGGCGCGTGTACGGCGCGATCGTCGGCGACAACGTCGTGCAGCTCGCGCAGCCGCTATCGGTGCGCAACCGGCTCGCCG
                       ||||||||||||||||||||||||||||||||||||||||||||||||||||||||||||||||||||||||||||||||||||||||||||||||||||||||||||||||||||||||
pBBRqseBC              CCGAGCTCGGCTTCTCGACCGAGCGCACCGAGCGCGGCGCGTGGCGCGTGTACGGCGCGATCGTCGGCGACAACGTCGTGCAGCTCGCGCAGCCGCTATCGGTGCGCAACCGGCTCGCCG

                         1090      1100      1110      1120      1130      1140      1150      1160      1170      1180      1190      1200
B.pseudomallei K96243  CGAGCGTCGCGCTGCGCACGCTCTGGCCGCTCATCGTGCTGCTGCCCTTCCTCGGCGCGGCCGTCTGGATGATCGTCGGGCGCGGGCTCGCGCCGCTGCAGCGCGTGACCCGCGCAGTCG
                       ||||||||||||||||||||||||||||||||||||||||||||||||||||||||||||||||||||||||||||||||||||||||||||||||||||||||||||||||||||||||
pBBRqseBC              CGAGCGTCGCGCTGCGCACGCTCTGGCCGCTCATCGTGCTGCTGCCCTTCCTCGGCGCGGCCGTCTGGATGATCGTCGGGCGCGGGCTCGCGCCGCTGCAGCGCGTGACCCGCGCAGTCG

                         1210      1220      1230      1240      1250      1260      1270      1280      1290      1300      1310      1320
B.pseudomallei K96243  AGGCACGCCGGCCCGAGGCGCTCGATCCGCTGCCCGATTCGCCGCTGCCGCTCGAGGTGCGTCCGCTCGTGCGCGCGCTCAACGGGCTGCTTGCACGGCTGTCGGCCGCGCTCGACACGC
                       ||||||||||||||||||||||||||||||||||||||||||||||||||||||||||||||||||||||||||||||||||||||||||||||||||||||||||||||||||||||||
pBBRqseBC              AGGCACGCCGGCCCGAGGCGCTCGATCCGCTGCCCGATTCGCCGCTGCCGCTCGAGGTGCGTCCGCTCGTGCGCGCGCTCAACGGGCTGCTTGCACGGCTGTCGGCCGCGCTCGACACGC

                         1330      1340      1350      1360      1370      1380      1390      1400      1410      1420      1430      1440
B.pseudomallei K96243  AAAAGGCGTTCGTCGCAGACGCCGCGCACGAGCTGCGCACGCCGCTTGCCGCCGTGCAGATTCAAGCGCAACTCGTCGCGCGTGCGCAGGACGACGTGTCGCGCCGCGAGGCGATCGTCG
                       ||||||||||||||||||||||||||||||||||||||||||||||||||||||||||||||||||||||||||||||||||||||||||||||||||||||||||||||||||||||||
pBBRqseBC              AAAAGGCGTTCGTCGCAGACGCCGCGCACGAGCTGCGCACGCCGCTTGCCGCCGTGCAGATTCAAGCGCAACTCGTCGCGCGTGCGCAGGACGACGTGTCGCGCCGCGAGGCGATCGTCG

                         1450      1460      1470      1480      1490      1500      1510      1520      1530      1540      1550      1560
B.pseudomallei K96243  ATCTGCAAAGCGGCGTCACGCGCGCGACGCGCCTCGCCGAGCAGTTGCTCGCGCTCGCGCGCGCCGAACCCGGCGACGCGACGGTGCGCGAGCGCGTCGATCTGCGCGCGATCGTCGAAG
                       ||||||||||||||||||||||||||||||||||||||||||||||||||||||||||||||||||||||||||||||||||||||||||||||||||||||||||||||||||||||||
pBBRqseBC              ATCTGCAAAGCGGCGTCACGCGCGCGACGCGCCTCGCCGAGCAGTTGCTCGCGCTCGCGCGCGCCGAACCCGGCGACGCGACGGTGCGCGAGCGCGTCGATCTGCGCGCGATCGTCGAAG

                         1570      1580      1590      1600      1610      1620      1630      1640      1650      1660      1670      1680
B.pseudomallei K96243  AGTGCGTCGCCGCACACGCGCCGCTCGCGCAGCGGCGCGACATCGATCTGGGCTTCGAGCGGGTCGAGGACGCGAGCGTGGACGCCGATCCCGCCGCGTTGCGCGTGATGTTCAACAACC
                       ||||||||||||||||||||||||||||||||||||||||||||||||||||||||||||||||||||||||||||||||||||||||||||||||||||||||||||||||||||||||
pBBRqseBC              AGTGCGTCGCCGCACACGCGCCGCTCGCGCAGCGGCGCGACATCGATCTGGGCTTCGAGCGGGTCGAGGACGCGAGCGTGGACGCCGATCCCGCCGCGTTGCGCGTGATGTTCAACAACC

                         1690      1700      1710      1720      1730      1740      1750      1760      1770      1780      1790      1800
B.pseudomallei K96243  TGCTCGACAACGCGGTGAAGTACACGCCGGCGGGCGGGCGCATCGACGTGTCGCTCACGCGCGGCGAAGGCGCGCGTGCGTGCGTGCAGATTGGCGACAGCGGCCCCGGCATCCCGGCCG
                       ||||||||||||||||||||||||||||||||||||||||||||||||||||||||||||||||||||||||||||||||||||||||||||||||||||||||||||||||||||||||
pBBRqseBC              TGCTCGACAACGCGGTGAAGTACACGCCGGCGGGCGGGCGCATCGACGTGTCGCTCACGCGCGGCGAAGGCGCGCGTGCGTGCGTGCAGATTGGCGACAGCGGCCCCGGCATCCCGGCCG

                         1810      1820      1830      1840      1850      1860      1870      1880      1890      1900      1910      1920
B.pseudomallei K96243  CCGAGCGCGAGCGCGTATTCGATCGTTTCTACCGCGACACGTCCGCGCGGGCGCGCACCGACGTCGCGGGCAGCGGCCTCGGTCTCGCGATCGTCAAGCGGGTGGCCGCGCAGCAGCGCG
                       ||||||||||||||||||||||||||||||||||||||||||||||||||||||||||||||||||||||||||||||||||||||||||||||||||||||||||||||||||||||||
pBBRqseBC              CCGAGCGCGAGCGCGTATTCGATCGTTTCTACCGCGACACGTCCGCGCGGGCGCGCACCGACGTCGCGGGCAGCGGCCTCGGTCTCGCGATCGTCAAGCGGGTGGCCGCGCAGCAGCGCG

                         1930      1940      1950      1960      1970      1980      1990
B.pseudomallei K96243  CGAGCGTGACGCTCGGCGAGGCGGCGGCGGGCGGGCTGCTCGTCAGCGTCTCGCTGCCGTGCGTGGGCCCCGCGTGA
                       |||||||||||||||||||||||||||||||||||||||||||||||||||||||||||||||||||||||||||||
pBBRqseBC              CGAGCGTGACGCTCGGCGAGGCGGCGGCGGGCGGGCTGCTCGTCAGCGTCTCGCTGCCGTGCGTGGGCCCCGCGTGA
```
