# Supplementary material for: Analysis of the role of the QseBC two-component sensory system in epinephrine-induced motility and intracellular replication of Burkholderia pseudomallei
Source: PLoS One. 2023 Feb 23;18(2):e0282098. doi: 10.1371/journal.pone.0282098 (PMC9949665; doi:10.1371/journal.pone.0282098)
Supplement: S2 Fig — QseB and QseC amino acid sequences from B. pseudomallei K96243 (UniProt accession numbers: Q63WT5, Q63WT4) was compared with those from B. mallei ATCC 23344 (UniProt accession numbers: A0A0H2WJH0, A0A0H2WIM0), B. thailandensis E264 (UniProt accession numbers: Q2T0S0, Q2T0R9), B. cepacia ATCC25416 (UniProt accession numbers: A0A806UVQ1, A0A806V178), B. cenocepacia ATCCJ2315 (UniProt accession numbers: B4EA15, B4EA14), B. multivorans ATCC17616 (UniProt accession numbers: A0A0H3KLT5, A0A0H3KH15), B. gladioli (UniProt accession numbers: A0A095FGQ9, A0A095FGJ4), B. dolosa AU0158 (UniProt accession numbers: A2W7S9, A2W7T0), B. glumae BGR1 (UniProt accession numbers: C5ACR6, C5ACR5) and E. coli O157:H7 (UniProt accession numbers: Q8XBS3, Q8X524). Residues in the periplasmic domain of E. coli O157:H7 QseC that are conserved across QseC homologues from diverse bacteria are highlighted in yellow. The symbols demonstrate the scale of conservation, whereby asterisk (*) indicates a fully conserved residue, colon (:) indicates conservation between groups of strongly similar properties and period (.) indicates conservation between groups of weakly similar properties. The functional domains were predicted using a Simple Modular Architecture Research Tool or SMART (http://smart.embl.de). The functional regions were highlighted in different colors (green, receiver domain; blue, transcriptional regulatory domain; grey, transmembrane region; orange, HAMP domain; pink, histidine kinase domain; brown, histidine kinase-like ATPase). (PDF) [file pone.0282098.s002.pdf]

**S2 Fig. Multiple protein sequence alignments of *B. pseudomallei* QseB and QseC relative to homologous proteins from other *Burkholderia* species and *E. coli* O157:H7.** QseB and QseC amino acid sequences from *B. pseudomallei* K96243 (UniProt accession numbers: Q63WT5, Q63WT4) was compared with those from *B. mallei* ATCC 23344 (UniProt accession numbers: A0A0H2WJH0, A0A0H2WIM0), *B. thailandensis* E264 (UniProt accession numbers: Q2T0S0, Q2T0R9), *B. cepacia* ATCC25416 (UniProt accession numbers: A0A806UVQ1, A0A806V178), *B. cenocepacia* ATCCJ2315 (UniProt accession numbers: B4EA15, B4EA14), *B. multivorans* ATCC17616 (UniProt accession numbers: A0A0H3KLT5, A0A0H3KH15), *B. gladioli* (UniProt accession numbers: A0A095FGQ9, A0A095FGJ4), *B. dolosa* AU0158 (UniProt accession numbers: A2W7S9, A2W7T0), *B. glumae* BGR1 (UniProt accession numbers: C5ACR6, C5ACR5) and *E. coli* O157:H7 (UniProt accession numbers: Q8XBS3, Q8X524). Residues in the periplasmic domain of *E. coli* O157:H7 QseC that are conserved across QseC homologues from diverse bacteria are highlighted in yellow. The symbols demonstrate the scale of conservation, whereby asterisk (*) indicates a fully conserved residue, colon (:) indicates conservation between groups of strongly similar properties and period (.) indicates conservation between groups of weakly similar properties. The functional domains were predicted using a Simple Modular Architecture Research Tool or SMART (http://smart.embl.de). The functional regions were highlighted in different colors (green, receiver domain; blue, transcriptional regulatory domain; grey, transmembrane region; orange, HAMP domain; pink, histidine kinase domain; brown, histidine kinase-like ATPase).

## QseB

```
B. pseudomallei      -------------------------------MRILLVEDDRMIAEGVRKALKADGCAVDW 29
B. mallei            -------------------------------MRILLVEDDRMIAEGVRKALKADGCAVDW 29
B. thailandensis     ----------MRARRRYNVRHLERMRRTFFRMRILLVEDDRMIADGVRKALKADGCAVDW 50
B. cepacia           -------------------------------MRILLVEDDRMIAEGVRKALRSDGFAVDW 29
B. cenocepacia       -------------------------------MRILLVEDDRMIAEGVRKALRSDGFAVDW 29
B. multivolans       -------------------------------MRILLVEDDRMIAEGVRKALRSDGFAVDW 29
B. gladioli          -------------------------------MRILLVEDDKMIAEGVRKALKADGFAVDW 29
B. dolosa            MRRVCGAASRTRDSRRYNGRHLSLVRWTPVPMRILLVEDDRMIAEGVRKALRSDGFAVDW 60
B. glumae            --------------------------MDADTMRILLVEDDRMIAEGVRKALKADGCAVDW 34
E. coli O157:H7      -------------------------------MRILLIEDDMLIGDGIKTGLSKMGFSVDW 29
                                                    *****:*** :*.:*::..*   * :***

B. pseudomallei      VQDGDAALTALGGEAYDLLLLDLGLPKRDGIDVLRTLRARGLALPVLILTARDAVADRVK 89
B. mallei            VQDGDAALTALGGEAYDLLLLDLGLPKRDGIDVLRTLRARGLALPVLILTARDAVADRVK 89
B. thailandensis     VQDGDAALTALGGEAYDLLLLDLGLPKRDGIDVLRTLRARGLALPVLILTARDAVADRVK 110
B. cepacia           VQDGDAALTALGGETYDLLLLDLGLPKRDGIDVLRTLRARGLALPVLIVTARDAVADRVK 89
B. cenocepacia       VQDGDAALTALGGETYDLLLLDLGLPKRDGIDVLRTLRGRGLALPVLIVTARDAVADRVK 89
B. multivolans       VQDGESALTALGGESYDLLLLDLGLPKRDGIDVLRTLRARGLSLPVLIVTARDAIADRVK 89
B. gladioli          VQDGEAALTAIGGETYDLMLLDLGLPKRDGVDVLRTLRSRGHALPVLIITARDAVSDRVK 89
B. dolosa            VQDGEAALTALGGEAYDLLLLDLGLPKRDGIDVLRTLRARGLALPVLIVTARDAVADRVK 120
B. glumae            VQDGEAALTALGGETYDLMLLDLGLPKRDGVEVLRTARARGHALPVLIITARDAVPDRVK 94
E. coli O157:H7      FTQGRQGKEALYSAPYDAVILDLTLPGMDGRDILREWREKGQREPVLILTARDALEERVE 89
                     . :*  .  *: .  ** ::*** **  ** ::**  * :*   ****:*****: :**:
```

**Receiver domain**

```
B. pseudomallei      GLDAGADDYLVKPFDLDELAARMRALIRRQSGRSESMIRHGALTLDPASHQVTLDGAPVA 149
B. mallei            GLDAGADDYLVKPFDLDELAARMRALIRRQSGRSESMIRHGALTLDPASHQVTLDGAPVA 149
B. thailandensis     GLDAGADDYLVKPFDLDELAARMRALIRRQSGRSESVIRHGALTLDPASHQVTLDGAPVA 170
B. cepacia           GLDAGADDYLVKPFDLDELGARMRALIRRQAGRSESLIRHGALTLDPASHQVTLDGAPVA 149
B. cenocepacia       GLDAGADDYLVKPFDLDELGARMRALIRRQAGRSESLIRHGALTLDPASHQVTLDGAPVA 149
B. multivolans       GLDAGADDYLVKPFDLDELGARMRALIRRQAGRSESLIRHGALTLDPASHQVTLDGAPVA 149
B. gladioli          GLDAGADDYLVKPFDLDELGARMRALIRRQSGRSESVIRHGALTLDPASHQVTLDGAAVA 149
B. dolosa            GLDAGADDYLVKPFDLDELGARMRALIRRQAGRSESLIRHGTLTLDPASHQVTLDGAPVA 180
B. glumae            GLDAGADDYLVKPFDLDELAARMRALIRRQAGRSESVIRHGALTLDPASHQVTLDGAPVA 154
E. coli O157:H7      GLRLGADDYLCKPFALIEVAARLEALMRRTNGQASNELRHGNVMLDPGKRIATLAGEPLT 149
                     **  ****** *** * *:.**:.**:**  *::.. :*** : ***..: .** *  ::

B. pseudomallei      LSAREFALLEALLARPGAVLSKSQLEEKMYGWGEEIGSNTVEVYIHALRKKLGSDLIRNV 209
B. mallei            LSAREFALLEALLARPGAVLSKSQLEEKMYGWGEEIGSNTVEVYIHALRKKLGSDLIRNV 209
B. thailandensis     LSAREFALLEALLARPGAVLSKSQLEEKMYGWGEEIGSNTVEVYIHALRKKLGSDLIRNV 230
B. cepacia           LSAREFALLEALLARPGAVLSKSQLEEKMYGWGEEIGSNTVEVYIHALRKKLGSDLIRNV 209
B. cenocepacia       LSAREFALLEALLARPGAVLSKSQLEEKMYGWGEEIGSNTVEVYIHALRKKLGSDLIRNV 209
B. multivolans       LSAREFALLEALLARPGAVLSKSQLEEKMYGWGEEIGSNTVEVYIHALRKKLGSDLIRNV 209
B. gladioli          LSAREFALLEALLARPGAVLSKSQLEEKMYGWGEEIGSNTVEVYIHALRKKLGSDLIRNV 209
B. dolosa            LSAREFALLEALLARPGAVLSKSQLEEKMYGWGEEIGSNTVEVYIHALRKKLGSDLIRNV 240
B. glumae            LSAREFALLEALLARPGAVLSKSQLEEKMYGWGEEIGSNTVEVYIHALRKKLGSDLIRNV 214
E. coli O157:H7      LKPKEFALLELLMRNAGRVLPRKLIEEKLYTWDEEVTSNAVEVHVHHLRRKLGSDFIRTV 209
                     *. :****** *: . * ** :. :***:* *.**: **:***::* **:*****:**.*
```

**Transcriptional regulatory domain**

```
B. pseudomallei      RGLGYMVVKEA 220
B. mallei            RGLGYMVVKEA 220
B. thailandensis     RGLGYMVVKEA 241
B. cepacia           RGLGYMVVKES 220
B. cenocepacia       RGLGYMVVKES 220
B. multivolans       RGLGYMVVKES 220
B. gladioli          RGLGYMVVKES 220
B. dolosa            RGLGYMVVKES 251
B. glumae            RGLGYMVVKEA 225
E. coli O157:H7      HGIGYTLGEK- 219
                     :*:** : ::
```

## QseC

```
B. pseudomallei       -----MRSIRHQLLIWLLAIVVAGVGLAGWMIYRQALAAANELFDYQLQQIAAALPSEPF 55
B. mallei             -----MRSIRHQLLIWLLAIVVAGVGLAGWMIYRQALAAANELFDYQLQQIAAALPSEPF 55
B. thailandensis      -----MRSIRHQLLIWLLAIVVAGVGLAGWMIYRQALAAANELFDYQLQQIAAALPSEPF 55
B. cepacia            -----MRSIRHQLLIWLLAIVVAGVGIAGWLIYRQALAEANELFDYQLQEIAAALPSEPF 55
B. cenocepacia        -----MRSIRHQLLIWLLAIVVAGVGAAGWLIYRQALAEANELFDYQLQEIAAALPSEPF 55
B. multivolans        -----MRSIRHQLLIWLLAIVVAGVSVAGWLIYRQALAEANELFDYQLQEIAAALPSEPF 55
B. gladioli           -----MRSIRHQLLIWLLAIVVAGVGAAGWLIYRQALAEANELFDYQLQAIAAALPSEPF 55
B. dolosa             -----MRSIRHQLLVWLLAIVVAGVGMAGWLIYRQALAEANELFDYQLQEIAAALPSEPF 55
B. glumae             -----MRSIRHQLLIWLLAIVVAGVGAAGWLIYRQALAEANELFDYQLQEIAAALPSEPF 55
E. coli O157:H7       MKFTQRLSLRVRLTLIFLILASVTWLLSSFVAWKQTTDNVDELFDTQLMLFAKRLSTLDL 60
                             *:* :* : :* :. .    :.:: ::*:   .:**** **  :*  * :  :
```

Transmembrane region

```
B. pseudomallei       SQVLGSQ-----------TN-GDEGIVIQIWNRNGVLMYFSHPRAPIAPR--AELGFS-- 99
B. mallei             SQVLGSQ-----------TN-GDEGIVIQIWNRNGVLMYFSHPRAPIAPR--AELGFS-- 99
B. thailandensis      SQVLGSQ-----------TN-GDEGIVIQIWNRNGVLMYFSHPRAPIAPR--AELGFS-- 99
B. cepacia            SQVFGSR-----------TN-GDEGIVIQIWNRNGVLMYFSHPRAPIAPR--AELGFS-- 99
B. cenocepacia        SQVFGSR-----------TN-GDEGIVIQIWNRNGVLMYFSHPRAPIAPR--AELGFS-- 99
B. multivolans        SQVFGSR-----------TN-GDEGIVIQIWNRNGVLMYFSHPRAPIAPR--AELGFS-- 99
B. gladioli           SQVFGSR-----------TN-GDEGIVIQIWNRNGGLMYFSHPRAPIAPR--AELGFS-- 99
B. dolosa             SQVFGSR-----------TN-GDEGIVIQIWNRNGVLMYFSHPRAPIAPR--AELGFS-- 99
B. glumae             SQVFGSR-----------TTGGDEGIVIQIWNRNGNLMYFSHPRAPIAPR--AELGFS-- 100
E. coli O157:H7       NEINAADRMAQTPNKLKHGHVDDDALTFAIFTHDGRMVLNDGDNGEDIPYSYQREGFADG 120
                      .:: .:               .*:.:.: *:.::* ::  .  ..   *    . **:
```

```
B. pseudomallei       TERTERGAWRVYGAIV--GDNVVQLAQPLSVRNRLAASVALRTLWPLIVLLPFLGAAVWM 157
B. mallei             TERTERGAWRVYGAIV--GDNVVQLAQPLSVRNRLAASVALRTLWPLIVLLPFLGAAVWM 157
B. thailandensis      TERTERGAWRVYGAIV--GDNVVQLAQPLSVRNRLAASVALRTLWPLIVLLPFLGAAVWM 157
B. cepacia            TERTDRGEWRVYGAIV--GDNVVQLAQPLSVRNRLAANVALRTLWPLIVLLPFLGAAVWV 157
B. cenocepacia        TERTDRGEWRVYGAIV--GDNVVQLAQPLSVRNRLAANVALRTLWPLIVPLPFLGAAAWG 157
B. multivolans        TERTDRGEWRVYGAIV--GDNVVQLAQPLSVRNRLAANVALRTLWPLIVLLPFLGAAVWM 157
B. gladioli           TERTDRGEWRVYGAIV--GDNVVQLAQPLSVRNRLAASVALRTVWPLILLLPFLGAAVWM 157
B. dolosa             TERTDRGEWRVYGAIV--GDNVVQLAQPLSVRNRLAANVALRTLWPLIVLLPFLGAAVWM 157
B. glumae             TEHTDRGEWRVYGAIV--GDNVVQLAQPVSVRNRLAASVALRTLWPLIVLLPFLGAAVWM 158
E. coli O157:H7       QLVGDKDQWRFVWMTSPDGKYRIVVGQEWEYREDMALAIVAGQLIPWLVALPVMLIIMMV 180
                          ::. **.       *.  : :.*  . *: :*  :.   : * :: **.:
```

Transmembrane region

```
B. pseudomallei       IVGRGLAPLQRVTRAVEARRPEALDPLPDSPLPLEVRPLVRALNGLLARLSAALDTQKAF 217
B. mallei             IVGRGLAPLQRVTRAVEARRPEALDPLPDSPLPLEVRPLVRALNGLLARLSAALDTQKAF 217
B. thailandensis      IVGRGLAPLQRVTRAVEARRPEALDPLPDSPLPLEVRPLVRALNGLLARLSAALDTQKAF 217
B. cepacia            IVGRGLAPLGRVTRAVEARRPEALDPLPDARLPLEVQPLVRALNGLLARLSAALDTQKAF 217
B. cenocepacia        IVGRGLAPLGRVARAVEARRPEALDPLPDARLPLEVQPLVRALNGLLARLSAALDTQKAF 217
B. multivolans        IVGRGLAPLGRVTRAVETRRPEALDPLPDARLPLEVQPLVRALNGLLARLSGALDTQKAF 217
B. gladioli           IVGRGMRPLRRVTRAVESRRPEALDPLPDNRLPQEVQPLVHALNGLLARLAAALDTQKAF 217
B. dolosa             IVGRGLAPLARVTHAVEARRPEALDPLPDARLPLEVQPLVRALNGLLARLSSALDTQKAF 217
B. glumae             IVGRGMRPLRRVTRAVESRRPEALDPLPDARLPLEVQPLVHALNGLLSRLAAALDTQKAF 218
E. coli O157:H7       LLGRELAPLNKLALALRMRDPDSEKPLNATGVPSEVRPLVESLNQLFARTHAMMVRERRF 240
                      ::** : ** ::: *:. * *:: .**    :* **:***.:** *::*  . :  :: *
```

HAMP domain

```
B. pseudomallei       VADAAHELRTPLAAVQIQAQLVARAQDD-VSRREAIVDLQSGVTRATRLAEQLLALARAE 276
B. mallei             VADAAHELRTPLAAVQIQAQLVARAQDD-VSRREAIVDLQSGVTRATRLAEQLLALARAE 276
B. thailandensis      VADAAHELRTPLAAVQIQAQLVARAQDD-ASRREAIVDLQSGVTRATRLAEQLLALARAE 276
B. cepacia            VADAAHELRTPLAAVQIQAQLVARAKDD-ASRREAVADLQDGVSRATRLTEQLLALARAE 276
B. cenocepacia        VADAAHELRTPLAAVQIQAQLVARAKDD-ASRREAIADLQDGVSRATRLTEQLLALARAE 276
B. multivolans        VADAAHELRTPLAAVQIQAQLVARAKDD-AARREALDDLQHGVTRATRLAEQLLALARAE 276
B. gladioli           VADAAHELRTPLAAVQIQAQLVARAQDD-ETRREALADLQSGVTRATRLAEQLLALARSE 276
B. dolosa             VADAAHELRTPLAAVQIQAQLVARAKDD-ASRREALDDLQQGVTRATRLAEQLLALARAE 276
B. glumae             VADAAHELRTPLAAVQIQAQLVARAQDD-ETRREALADLQNGVTRATRLAEQLLALARSE 277
E. coli O157:H7       TSDAAHELRSPLTALKVQTEVAQLSDDDPQARKKALLQLHSGIDRATRLVDQLLTLSRLD 300
                      .:*******:**:*:::*:::.  :.**  :*::*: :*: *: *****.:***:*:* :
```

Histidine kinase domain

```
B. pseudomallei       PGDAT-VRERVDLRAIVEECVAAHAPLAQRRDIDLGFERVED-ASVDADPAALRVMFNNL 334
B. mallei             PGDAT-VRERVDLRAIVEECVAAHAPLAQRRDIDLGFERVED-ASVDADPAALRVMFNNL 334
B. thailandensis      PGDAT-VRERVDLRAIVEECVAAHAPLAQRRDIDLGFEQLED-ASVDADPAALRVMFNNL 334
B. cepacia            PDGAT-MREPVDLQALLAECVAALAPLAQRRDIDLGFEETHA-ASVVADVGALRVMFGNL 334
B. cenocepacia        PDGAT-MREPVDLQALLAECVAAHAPLAQRRDIDLGFEETRA-ASVVADVGALRVMFGNL 334
B. multivolans        PDAGA-VREPVDLQTLLAECVAAHAPLAQRRNIDLGFEETRA-ATVVADVGALRVMFGNL 334
B. gladioli           PDGAT-VREPVALADVLAHCVSAQAVVAQKRNIDLGIEEAQP-AIIDADIGALRVMLNNV 334
B. dolosa             PDGAT-VREPVDLHALLAECVAAYAPLAQRRGIDLGFEEARA-ATVVADVGALRVMFGNL 334
B. glumae             PDGAG-VIEPVALDEVLAGCVSAYAIVAQKRGIDLGIEATEP-AVIDADIGALRVMINNV 335
E. coli O157:H7       SLDNLQDVAEIPLEDLLQSSVMDIYHTAQQAKIDVRLTLNVQGIKRTGQPLLLSLLVRNL 360
                                :  *  ::  .*      **:  **: :          .:   * ::. *:

B. pseudomallei       LDNAVKYTPAGGRIDVSLTRGEGARACVQIGDSGPGIPAAERERVFDRFYRDTSARARTD 394
B. mallei             LDNAVKYTPAGGRIDVSLTRGEGARACVQIGDSGPGIPAAERERVFDRFYRDTSARARTD 394
B. thailandensis      LDNAVKYTPPGGRIDVSLTRGDGARACVQIGDSGPGIAAAERERVFDRFYRDTSARARTD 394
B. cepacia            LDNAVKYTPDGGRIDVSLTRDAAGRVCVQIGDSGPGIPADERERVFDRFYRDSSARARTD 394
B. cenocepacia        LDNAVKYTPDGGRIDVSLTRDAAGRACVQIGDSGPGIPADERERVFDRFYRDSTARARTD 394
B. multivolans        LDNAVKYTPDGGRIDVSLTRDAAGRACVQIGDSGPGIPAEERERVFDRFYRDSSARARTD 394
B. gladioli           LDNAVKYTPDGGRIDVSLSFD-EGRALVRVADSGPGIPPEERERVFDRFYRDSSARMRTD 393
B. dolosa             LDNAVKYTPDGGRIDVSLTRDAAGRACVQIGDSGPGIPADERERVFDRFYRDSSARARTD 394
B. glumae             LDNAVKYTPDGGRIDVSLGFAPDGPALVRIADSGPGIPPDERERVFDRFYRDSSARARTD 395
E. coli O157:H7       LDNAVRYSPQGSVVDVTLNAD-----NFIVRDNGPGVTPEALARIGERFYRPPGQ----T 411
                      *****:*:* *. :**:*         . : *.***:      *: :****
```

**Histidine kinase-like ATPases**

```
B. pseudomallei       VAGSGLGLAIVKRVAAQQRASVTLGEAAAGGLLVSVSLPCVGPA---------- 438
B. mallei             VAGSGLGLAIVKRVAAQQRASVTLGEAAAGGLLVSVSLPCAGPA---------- 438
B. thailandensis      VSGSGLGLAIVKRVAAQQRATVTLGEAAAGGLLVSVSLPRAGDA---------- 438
B. cepacia            VSGSGLGLAIVKRVAAQQGATVSLGDAAAGGLLVSVVFRDAEMPAEAPQPSESA 448
B. cenocepacia        VSGSGLGLAIVKRVAAQQGATVSLGDAAAGGLLVSVVFRDAEMPAPASQPSETV 448
B. multivolans        VSGSGLGLAIVKRVAAQQGATVSLGDAAAGGLLVSVVFRDAEMPAQAPQQPASV 448
B. gladioli           VSGSGLGLAIVKRVAAQQRATVTLGESPAGGLLVEIAFPGARLA---------- 437
B. dolosa             VSGSGLGLAIVKRVAAQQGATVSLGDAAAGGLLVSVVFRDAQLPADEAQSSEFA 448
B. glumae             VSGSGLGLAIVKRVAAQQGAVVTLGESDAGGLLVTLAFAAARRP---------- 439
E. coli O157:H7       ATGSGLGLSIVQRIAKLHGMNVEFGNAEQGGFEAKVSW---------------- 449
                      .:******:**:*:*  :   * :*::  **: . :
```
