# Supplementary figures and images for: Analysis of the role of the QseBC two-component sensory system in epinephrine-induced motility and intracellular replication of Burkholderia pseudomallei
Source: PLoS One. 2023 Feb 23;18(2):e0282098. doi: 10.1371/journal.pone.0282098 (PMC9949665; doi:10.1371/journal.pone.0282098)

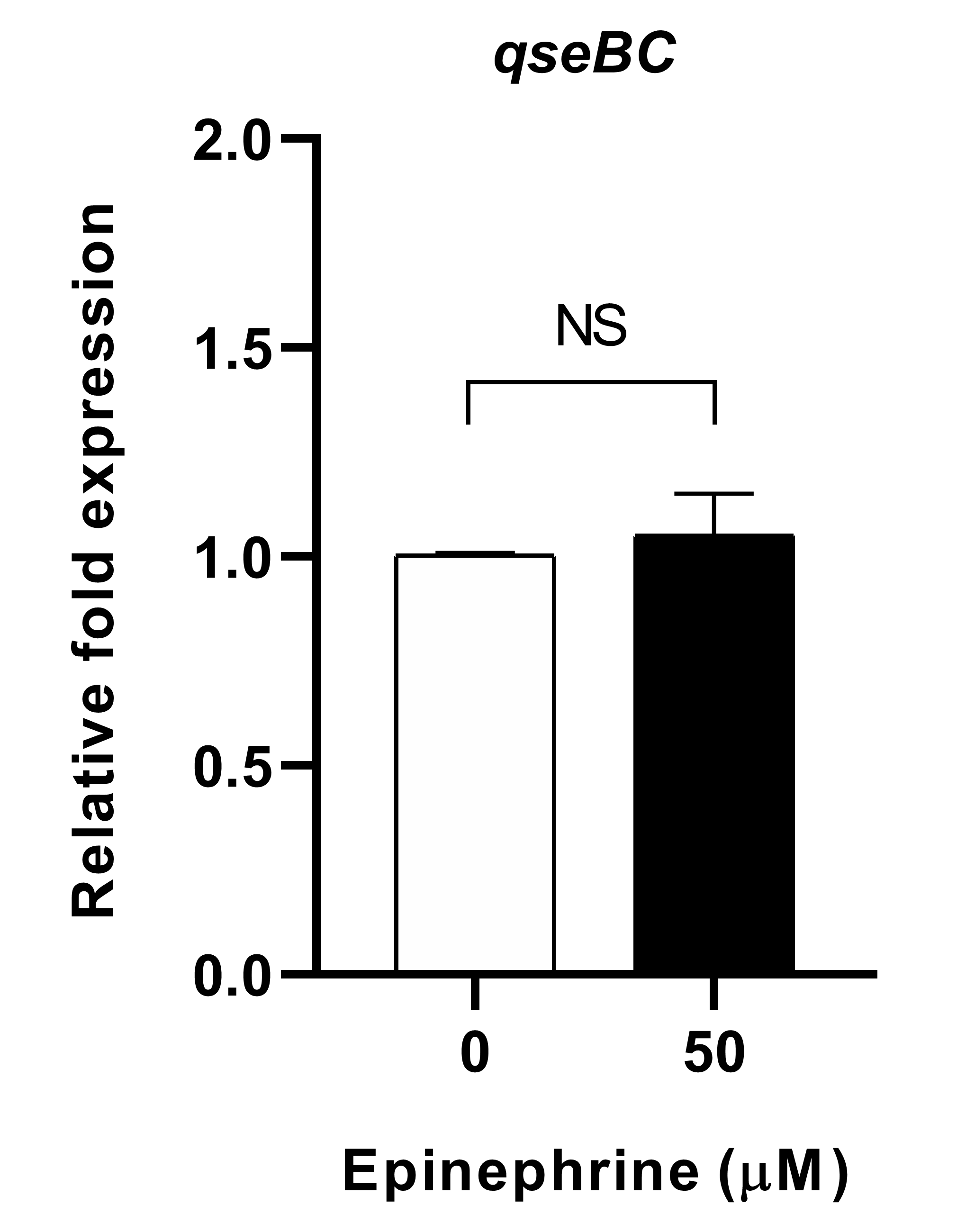

Supplement: S3 Fig — B. pseudomallei wild-type K96243 was grown in LB medium with or without 50μM epinephrine. The transcription of qseBC was analysed by real-time reverse transcriptase PCR. Three independent experiments were performed. Error bars represent the standard error of the mean. NS indicates no significant difference following a student’s t-test. (TIF) [file pone.0282098.s003.tif]

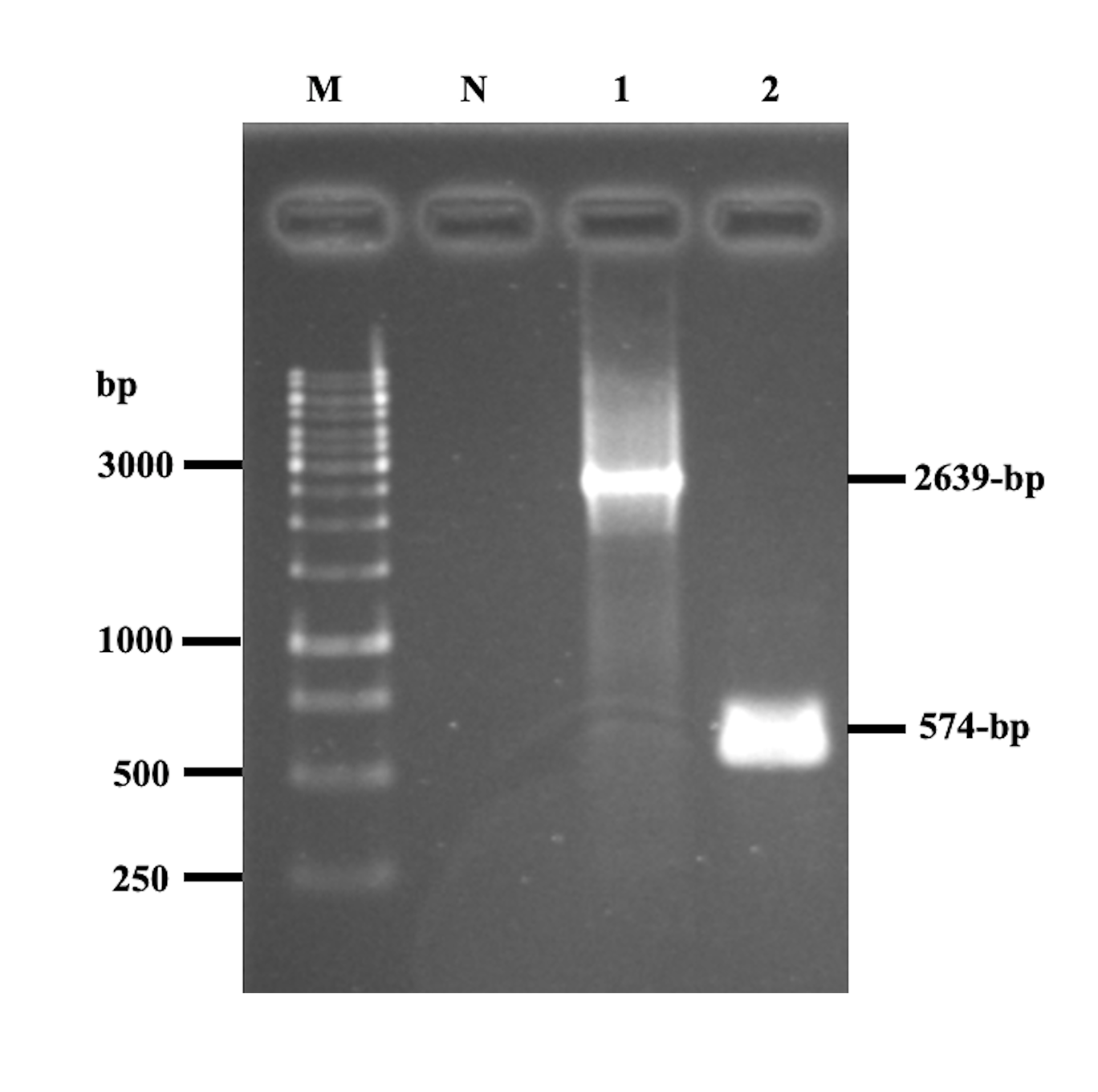

Supplement: S4 Fig — PCR analysis of B. pseudomallei wild-type K96243 compared with the ΔqseBC mutant using primers BPSL0806-F and BPSL0807-R. The amplicon predicted to be generated from B. pseudomallei wild-type is 2639-bp (lane 1) whereas for the ΔqseBC mutant it is 574-bp (lane 2). Lane M and N represent 1 kb DNA ladder and negative control, respectively. (TIFF) [file pone.0282098.s004.tiff]

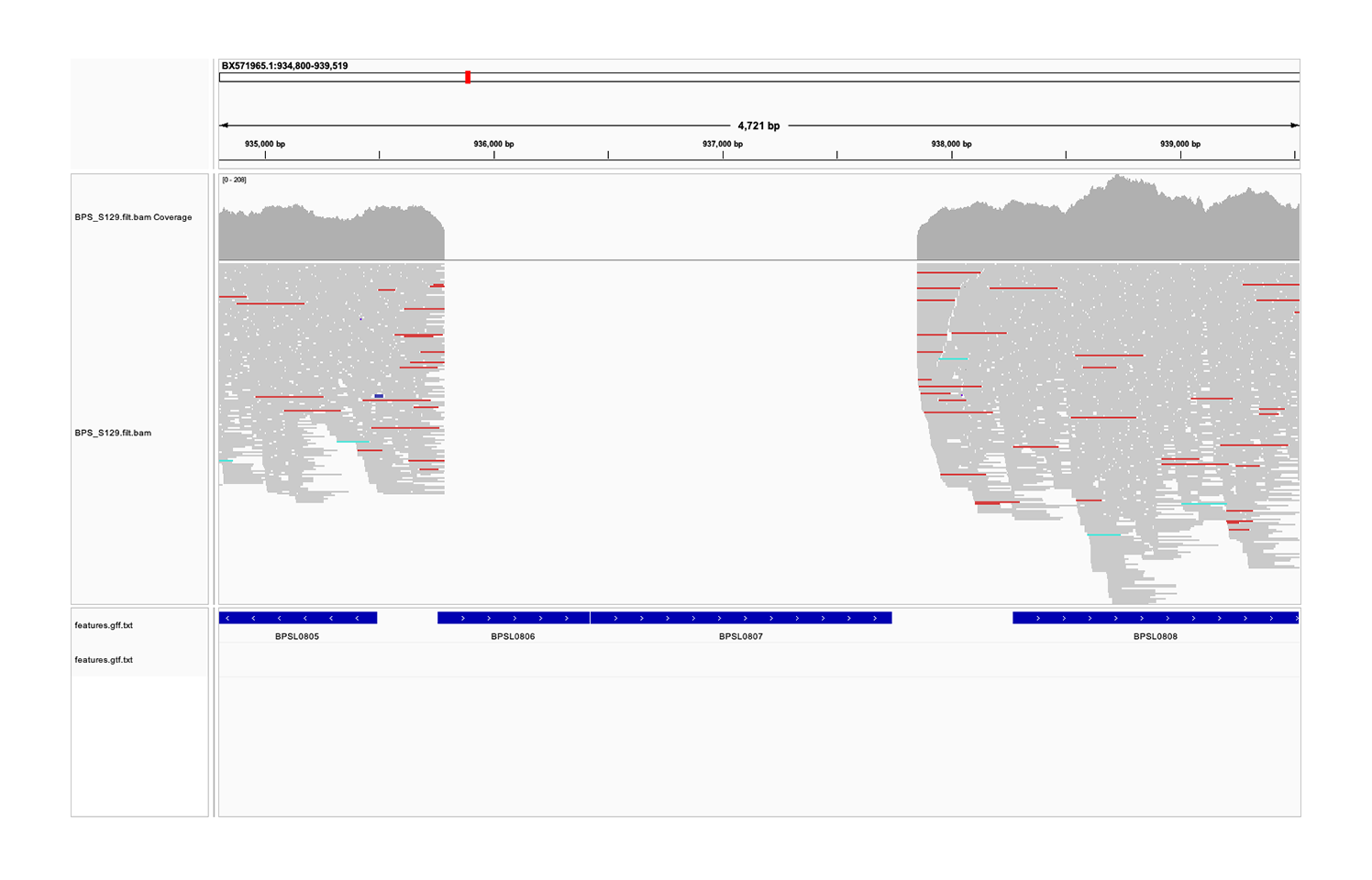

Supplement: S5 Fig — The sequence genomes of K96243 and ΔqseBC mutant were compared with each other in chromosome 1. Deletions between bpsl0806 and bpsl0807 in B. pseudomallei ΔqseBC mutant were shown. There were no effects on adjacent genes. (TIFF) [file pone.0282098.s005.tiff]

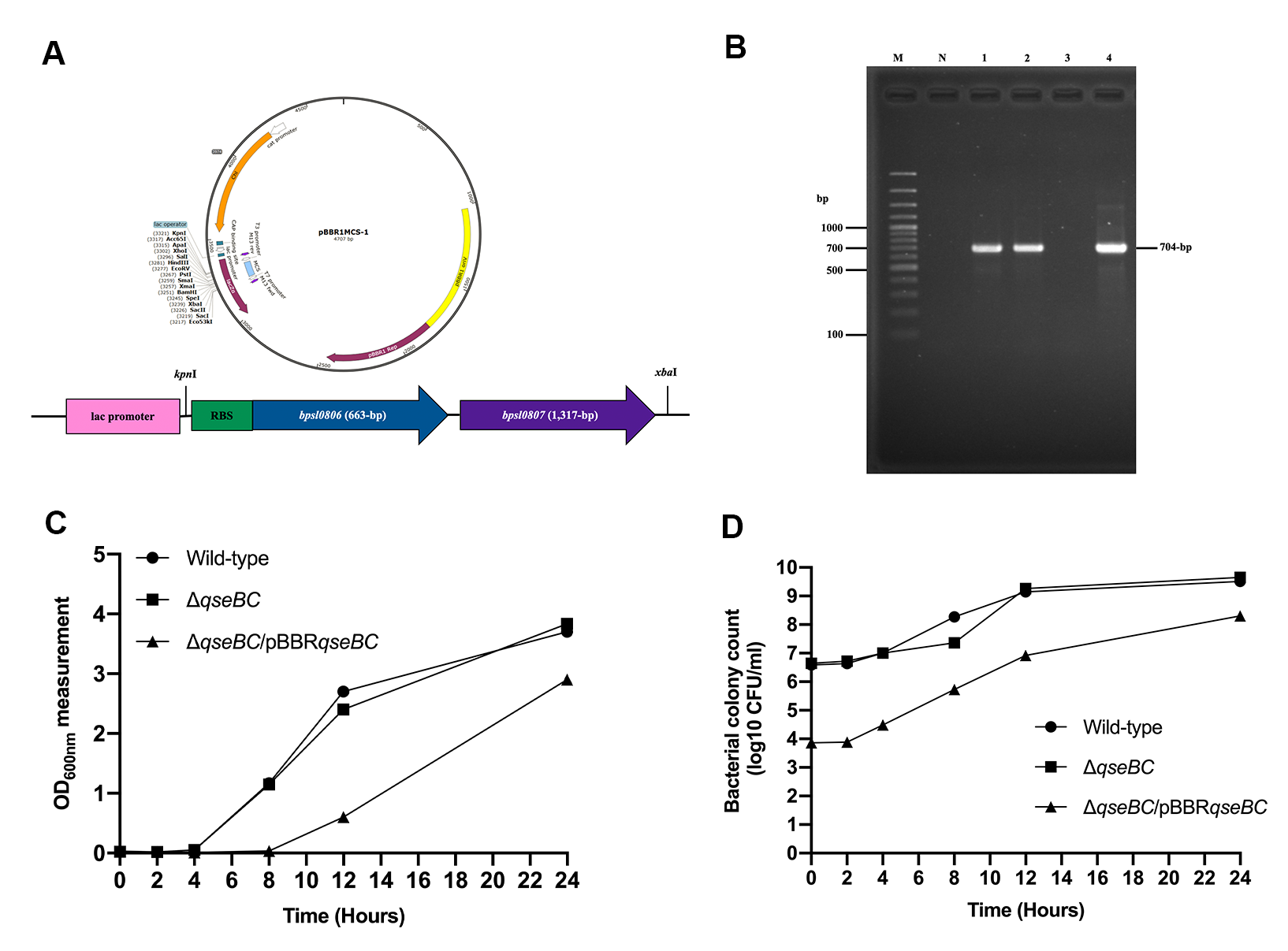

Supplement: S6 Fig — (A) Schematic representation of generation of plasmid pBBRqseBC. (B) RT-PCR analysis of qseBC expression using B. pseudomallei wild-type K96243 cDNA (lane 2), ΔqseBC mutant cDNA (lane 3) and ΔqseBC/pBBRqseBC cDNA (lane 4). Positive control using gDNA wild-type is shown in lane 1. Lanes M and N represent 100-bp DNA ladder and negative control, respectively. B. pseudomallei wild-type K96243, ΔqseBC mutant and ΔqseBC/pBBRqseBC complemented strains were grown in LB medium and bacterial cells were collected at 2, 4, 8, 12 and 24 h. for OD measurement (C) and colony count (D). Data represents the mean of triplicate determinations. (TIF) [file pone.0282098.s006.tif]
