# Supplementary material for: Analysis of the role of the QseBC two-component sensory system in epinephrine-induced motility and intracellular replication of Burkholderia pseudomallei
Source: PLoS One. 2023 Feb 23;18(2):e0282098. doi: 10.1371/journal.pone.0282098 (PMC9949665; doi:10.1371/journal.pone.0282098)
Supplement: S1 Table — (PDF) [file pone.0282098.s007.pdf]

**S1 Table. Bacterial strains and plasmids used in this study.**

| Strains or plasmids | Characteristics | Reference |
|---|---|---|
| **Strains** | | |
| *E. coli* DH5 alpha | $F^{-}$ $\phi$80*lacZ*△M15△(*lacZYA-argF*) *U169 deoR recA1 endA1 hsdR17* ($r_K^-$ $m_K^+$) *phoA supE44* | [1] |
| *E. coli* RHO3 | $Km^S$; SM10(λpir) △*asd*::*FRT* △*aphA*::*FRT* | [2] |
| *B. pseudomallei* K96243 | Human clinical isolate | [3] |
| *B. pseudomallei* Δ*qseBC* mutant | △*qseBC* mutant in strain K96243 | This study |
| *B. pseudomallei* Δ*qseBC*/pBBR*qseBC* | △*qseBC* mutant carrying pBBR*qseBC*; $Cm^r$ | This study |
| *B. pseudomallei bsaZ* mutant | Insertion *bipD*::pDM4 in strain 10276 | [4] |
| **Plasmids** | | |
| pGEM-T easy | TA cloning vector | Promega |
| pExKm5 | $Km^R$; pEXKm4 with *B. pseudomallei* optimized *sacB* gene region from pMo130 | [2] |
| pBADSce | $Zeo^R$; I-SceI expression vector with pRO1600(Ts) replicon | [2] |
| pBBR1MCS-1 | Broad host range plasmid, $Cm^R$ | [5] |

**S1 Table Supplemental References**


1. Hanahan D. Techniques for transformation of *E. coli*. DNA cloning: A practical approach. 11985. p. 109-35.
2. Lopez CM, Rholl DA, Trunck LA, Schweizer HP. Versatile dual-technology system for markerless allele replacement in *Burkholderia pseudomallei*. Appl Environ Microbiol. 2009;75(20):6496-503. Epub 2009/08/25. doi: 10.1128/AEM.01669-09. PubMed PMID: 19700544; PubMed Central PMCID: PMC2765137.
3. Holden MT, Titball RW, Peacock SJ, Cerdeno-Tarraga AM, Atkins T, Crossman LC, et al. Genomic plasticity of the causative agent of melioidosis, *Burkholderia pseudomallei*. Proc Natl Acad Sci U S A. 2004;101(39):14240-5. Epub 2004/09/21. doi: 10.1073/pnas.0403302101. PubMed PMID: 15377794; PubMed Central PMCID: PMC521101.
4. Stevens MP, Wood MW, Taylor LA, Monaghan P, Hawes P, Jones PW, et al. An Inv/Mxi-Spa-like type III protein secretion system in *Burkholderia pseudomallei* modulates intracellular behaviour of the pathogen. Mol Microbiol. 2002;46(3):649-59. doi: 10.1046/j.1365-2958.2002.03190.x. PubMed PMID: 12410823.
5. Obranic S, Babic F, Maravic-Vlahovicek G. Improvement of pBBR1MCS plasmids, a very useful series of broad-host-range cloning vectors. Plasmid. 2013;70(2):263-7. Epub 2013/04/16. doi: 10.1016/j.plasmid.2013.04.001. PubMed PMID: 23583732.
