# Supplementary material for: Analysis of the role of the QseBC two-component sensory system in epinephrine-induced motility and intracellular replication of Burkholderia pseudomallei
Source: PLoS One. 2023 Feb 23;18(2):e0282098. doi: 10.1371/journal.pone.0282098 (PMC9949665; doi:10.1371/journal.pone.0282098)
Supplement: S2 Table — (PDF) [file pone.0282098.s008.pdf]

S2 Table. List of primers used in this study.

Primer name	Oligonucleotide sequences (5'-3')	Amplicon size	Reference
BPSL0806-F	AATGCGGGAGAACGGAAGG	201-bp	This study
BPSL0806-R	CGATCGTCTTCAACCAGCAA		
BPSL0807-F	TTGCTGGTTGAAGACGATCGGATCCGTGGTTTGGCGATTC	393-bp	This study
BPSL0807-R	CGTGCAAGGATTCGGGTAGT		
Com_BPSL0806-F	TTAAGGTACCAGGAGGTTGCCATCATGCGGATATTGCTGGTTGAAGA	2017-bp	This study
Com_BPSL0807-R	TTATTCTAGATCACGCGGGGCCCACGCACG		
16s rRNA-F	AGACACGGCCCAGACTCCTAC	321-bp	[1]
16s rRNA-R	CAGTCACCAATGCAGTTCCCA		
fliC-F	ATGCAAACGCAGATCAACGG	338-bp	[2]
fliC-R	GACATGCTTTGCGTGAGGTC		
qseBC-F	ATGCGGATATTGCTGGTTGAAG	704-bp	This study
qseBC-R	AGCAGCCAGATCAGCAATTG		
*qseBC*_qRT-F	AGCCAGCTCGAGGAGAAGAT	287-bp	This study
*qseBC*_qRT-R	TGCTGCAGCTGATAGTCGAA		

S2 Table Supplemental References

1. Brett PJ, Deshazer D, Woods DE. Characterization of *Burkholderia pseudomallei* and *Burkholderia pseudomallei*-like strains. Epidemiol Infect. 1997;118(2):137-48. Epub 1997/04/01. doi: 10.1017/s095026889600739x. PubMed PMID: 9129590; PubMed Central PMCID: PMC2808781.
2. Intarak N, Muangsombut V, Vattanaviboon P, Stevens MP, Korbsrisate S. Growth, motility and resistance to oxidative stress of the melioidosis pathogen *Burkholderia pseudomallei* are enhanced by epinephrine. Pathog Dis. 2014;72(1):24-31. Epub 2014/04/23. doi: 10.1111/2049-632X.12181. PubMed PMID: 24753312.
