# Supplementary material for: Analysis of the role of the QseBC two-component sensory system in epinephrine-induced motility and intracellular replication of Burkholderia pseudomallei
Source: PLoS One. 2023 Feb 23;18(2):e0282098. doi: 10.1371/journal.pone.0282098 (PMC9949665; doi:10.1371/journal.pone.0282098)
Supplement: S3 Table — (PDF) [file pone.0282098.s009.pdf]

S3 Table. Identity and similarity of homologues of QseB and QseC across *Burkholderia* species.

Organism	UniProt Accession Number	Length (aa)	Identity* (%)	Similarity* (%)	Protein name
***B. pseudomallei* QseB (Q63WT5)**					
B. mallei ATCC23344	A0A0H2WJH0	220	100.00	100.00	DNA-binding response regulator
B. thailandensis E264	Q2T0S0	241	90.46	90.87	DNA-binding response regulator
B. cepacia ATCC25416	A0A806UVQ1	220	95.91	97.27	XRE family transcriptional regulator
B. cenocepacia ATCCJ2315	B4EA15	220	95.45	97.27	Two-component regulatory system, response regulator protein
B. multivorans ATCC17616	A0A0H3KLT5	220	94.09	96.36	OmpR family two-component system response regulator
B. gladioli	A0A095FGQ9	220	93.18	95.91	DNA-binding response regulator
B. dolosa AU0158	A2W7S9	251	83.67	85.26	DNA-binding response regulator
B. glumae BGR1	C5ACR6	225	92.89	95.11	DNA-binding response regulator
***B. pseudomallei* QseC (Q63WT4)**					
B. mallei ATCC23344	A0A0H2WIM0	438	99.77	100.00	Histidine kinase
B. thailandensis E264	Q2T0R9	438	97.49	98.63	Histidine kinase
B. cepacia ATCC25416	A0A806V178	448	84.60	88.62	Histidine kinase
B. cenocepacia ATCCJ2315	B4EA14	448	84.38	88.39	Histidine kinase
B. multivorans ATCC17616	A0A0H3KH15	448	83.93	88.17	Histidine kinase
B. gladioli	A0A095FGJ4	437	82.19	88.13	Histidine kinase
B. dolosa AU0158	A2W7T0	448	84.60	88.84	Histidine kinase
B. glumae BGR1	C5ACR5	439	80.41	85.65	Histidine kinase

* Calculation of percentage identity and similarity of QseB and QseC homologues across *Burkholderia* species relative to the proteins from *B. pseudomallei* K96243 was performed using Ident and Sim software (https://www.bioinformatics.org/sms2/ident_sim.html).
